# Supplementary material for: Translation reinitiation after uORFs does not fully protect mRNAs from nonsense-mediated decay
Source: RNA. 2023 Jun;29(6):735–44. doi: 10.1261/rna.079525.122 (PMC10187673; doi:10.1261/rna.079525.122)
Supplement: Supplemental Material [file supp_29_6_735__DC1.html]

Translation re-initiation after uORFs does not fully protect mRNAs from nonsense-mediated decay — Translation reinitiation after uORFs does not fully protect mRNAs from nonsense-mediated decay — Supplemental Material 

# Translation reinitiation after uORFs does not fully protect mRNAs from nonsense-mediated decay

## Supplemental Material

- Supplemental\_Material.pdf
- Supplemental\_Table\_1\_and2.xlsx
- Supplemental\_Table\_3.xlsx
