# Supplementary material for: Translation reinitiation after uORFs does not fully protect mRNAs from nonsense-mediated decay
Source: RNA. 2023 Jun;29(6):735–44. doi: 10.1261/rna.079525.122 (PMC10187673; doi:10.1261/rna.079525.122)
Supplement: Supplemental Material [file supp_079525.122_Supplemental_Material.pdf]

SUPPLEMENTAL MATERIAL

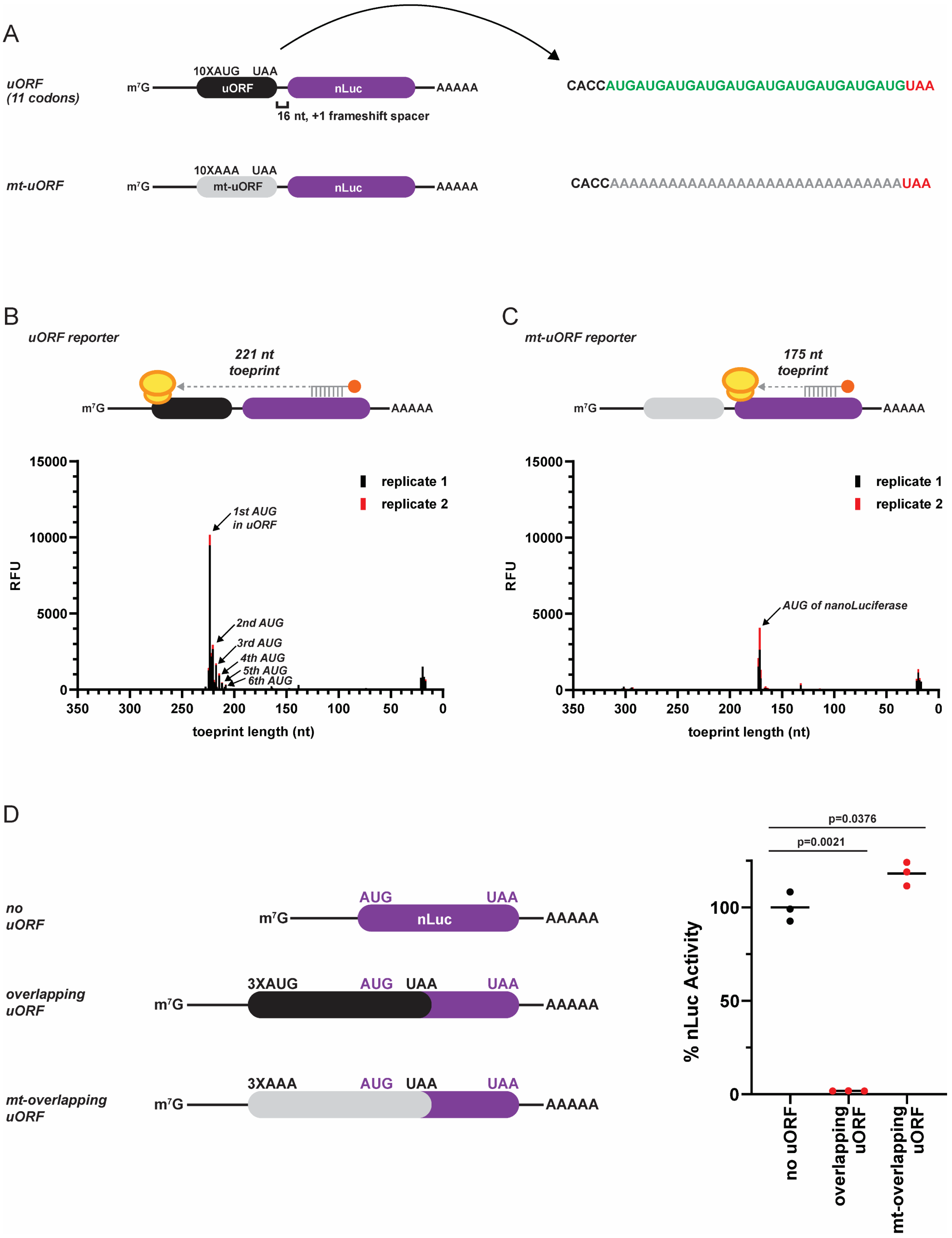

Supplemental Figure 1. Confirming the efficacy of designed uORFs to sequester all scanning initiation complexes *in vitro* and in HeLa cells. A) Design of re-initiation specific nanoLuciferase (nLuc) reporters harboring an uORF encoded by 10 consecutive AUG start

codons (uORF) or AAA codons (mt-uORF). A 16 nt spacer between the uORF and nLuc ORF allows specific detection of re-initiation. B-C) Ribosome toeprinting of lactimidomycin inhibited 80S ribosomes after start codon recognition on (B) small uORF nLuc reporter mRNA and (C) mutated uORF nLuc reporter mRNA from *in vitro* translation. Signal from duplicate samples is shown in black and red. D) Response of nLuc reporters that harbor an overlapping 3XAUG start codon uORF in HeLa cells. Here, the stop codon of the uORF (black) is downstream and out-of-frame of the start codon of nLuc (purple). Thus, active nLuc is only produced if a 43S PIC scans through the overlapping uORF (“leaky scanning”) and initiates at the start codon of nLuc. n=3 biological replicates. Bar represents the mean. Comparisons were made using a two-tailed unpaired *t*-test with Welch’s correction.

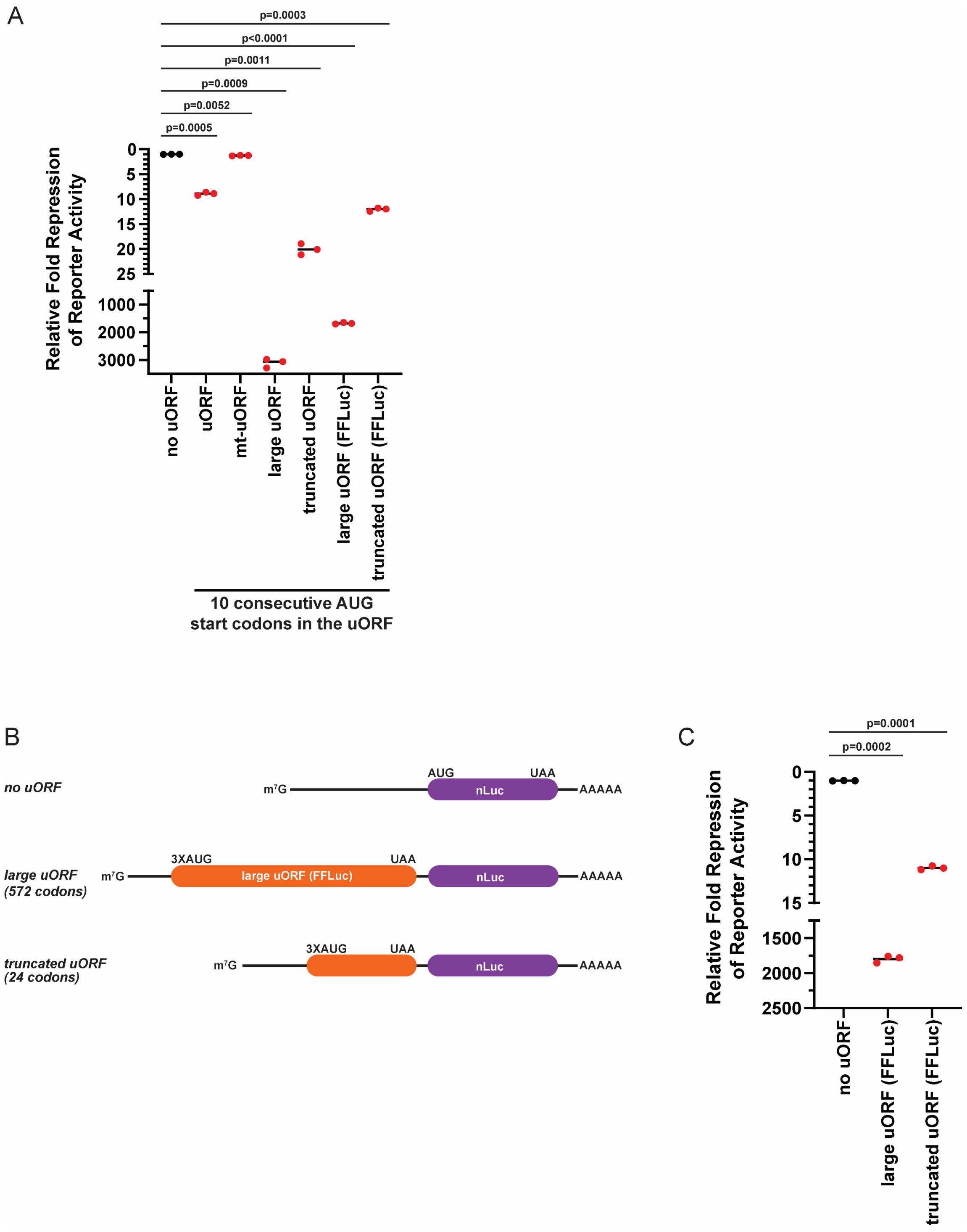

Supplemental Figure 2. Less translation re-initiation after large uORFs is not strictly uORF sequence specific. A) Response of nLuc reporters that harbor a small, mutant, large, or truncated uORF from *in vitro* translation. All uORFs had 10 consecutive AUG start codons as described in **Supplemental Figure 1A** (instead of the three AUG start codons in perfect Kozak

context in **Fig. 1**). Large and truncated uORFs using the FFLuc sequence are clearly labeled from those that used the original HT-GFP sequence. n=3 biological replicates. Bar represents the mean. Comparisons were made using a two-tailed unpaired *t*-test with Welch's correction. B) The large uORF sequence described in **Fig. 1** was switched from HT-GFP to Firefly Luciferase (FFLuc). Three AUG start codons in perfect Kozak context were used to trap all scanning initiation complexes at the uORF. C) Response of nLuc reporters that harbor a large (FFLuc) or truncated uORF (FFLuc) from *in vitro* translation. Similar repression and rescue were seen here with FFLuc as the large uORF as observed with HT-GFP sequence in **Fig. 2**. n=3 biological replicates. Bar represents the mean. Comparisons were made using a two-tailed unpaired *t*-test with Welch's correction.

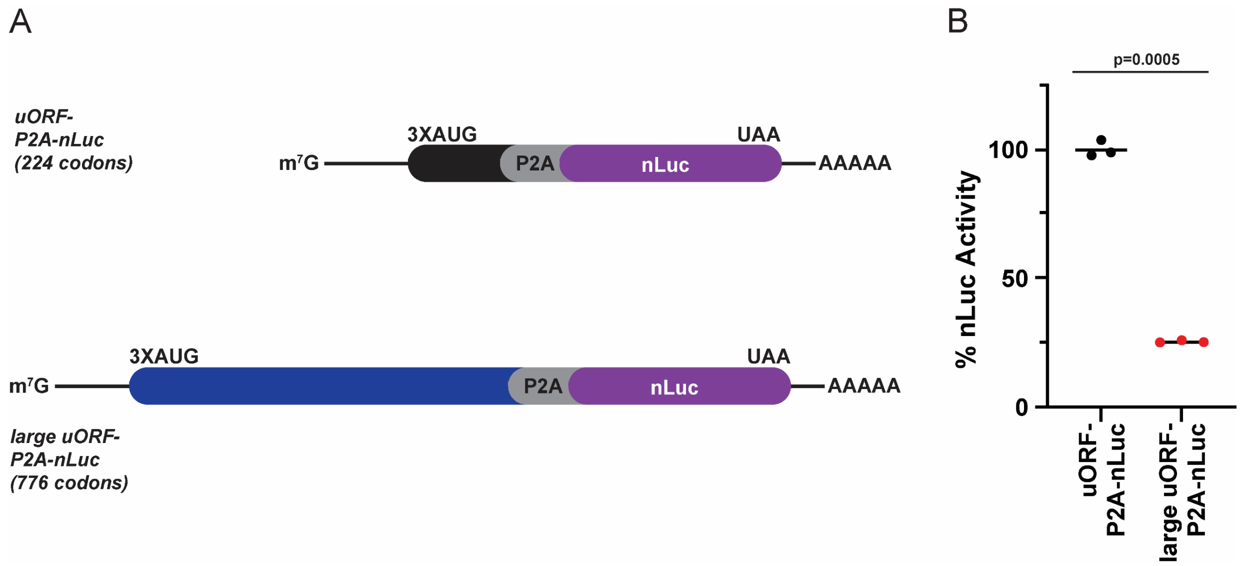

Supplemental Figure 3. Small and large uORF P2A fusion reporters to test translation efficiency *in vitro*. A) Insertion of the P2A "ribosome skipping motif" (gray) was used to assess the relative translation efficiency of reporters that harbored a 3XAUG start codon sequence without and with the large HT-GFP (blue) sequence upstream of the nLuc coding sequence (purple). B) Relative nLuc activity of the small and larger uORF P2A fusion reporters from *in vitro* translation. n=3 biological replicates. Bar represents the mean. Comparisons were made using a two-tailed unpaired *t*-test with Welch's correction.

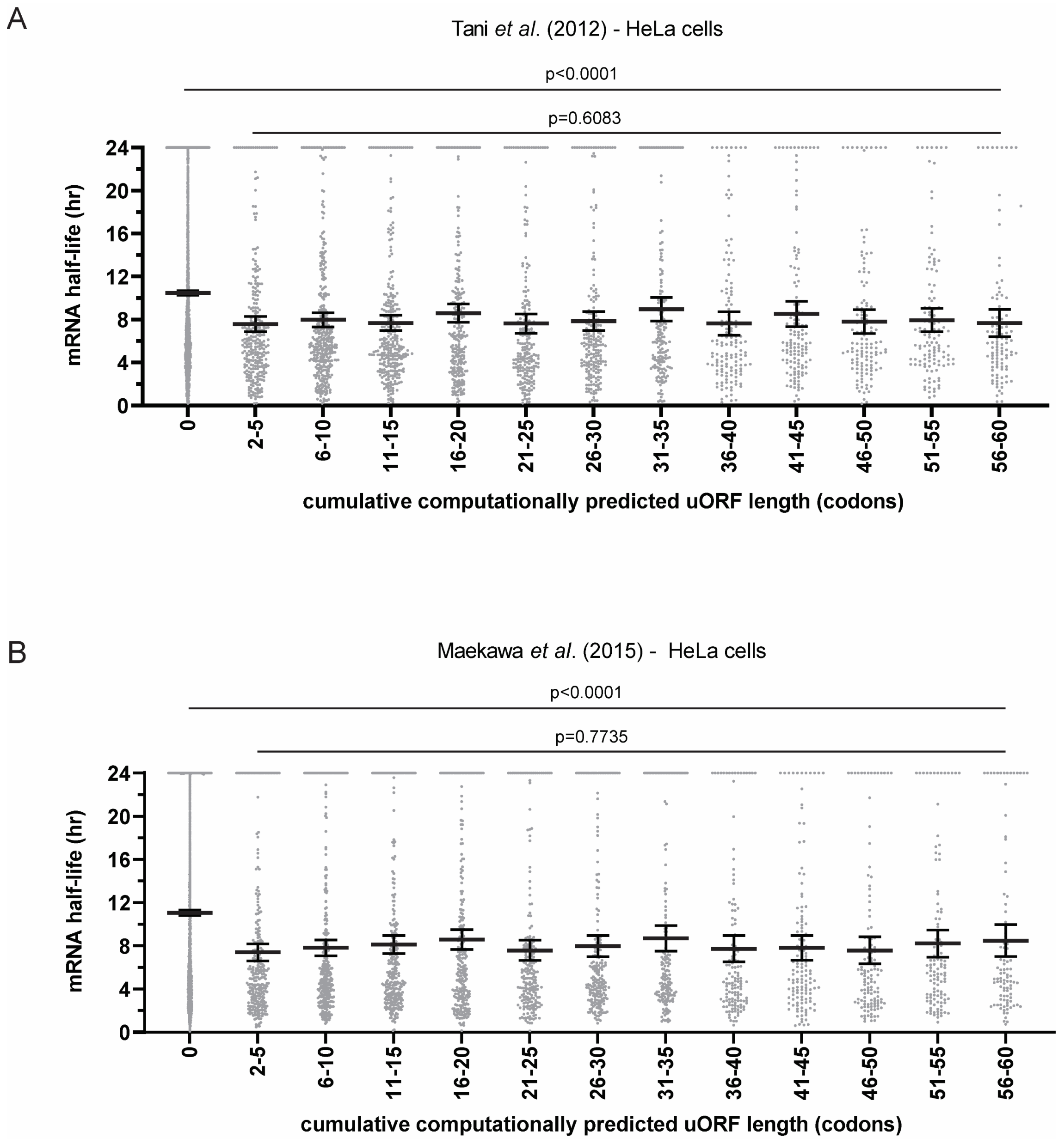

Supplemental Figure 4. uORF length has minimal effect on mRNA stability in cells. A-B) Transcriptome-wide comparison of cumulative computationally predicted uORF length and mRNA half-life from Tani *et al*. (2012) (A) and Maekawa *et al*. (2015) (B). Bar represents the mean ± 95% confidence interval. One-way Welch's ANOVA was used to compare between cumulative uORF length bins.

Sequences of reporters used in this study
The plasmid backbone, reporter name, and restriction sites are given for each construct. For reference, nanoLuciferase (nLuc) is always highlighted in pink. The multiple start sites for the uORF and single stop codon are highlighted in yellow and red, respectively. The HT-GFP sequence, FFLuc sequence and IRES sequences are highlighted in green, blue, and turquoise, respectively. The unstructured CAA repeats are in lowercase. The P2A sequence is highlighted in gray. The strong hairpins used upstream of the IRES sequences is in **bold**. For the pTet-Off All-In-One (pTet-Off AIO) plasmids, the tight TRE promotor is shown in purple and the intron is underlined.

pcDNA3.1+/no uORF nLuc (SacI/XbaI)
GAGCTCTCTGGCTAACTAGAGAACCCACTGCTTACTGGCTTATCGAAATTAATACGACTCA
CTATAGGcaacaacaacaacaacaacaacaacaacaacaacaacaacaacaacaacaacaacaacaacaacaacaac
aaATGGTCTTCACACTCGAAGATTTCGTTGGGGACTGGCGACAGACAGCCGGCTACAACCT
GGACCAAGTCCTTGAACAGGGAGGTGTGTCCAGTTTGTTTCAGAATCTCGGGGTGTCCGT
AACTCCGATCCAAAGGATTGTCCTGAGCGGTGAAAATGGGCTGAAGATCGACATCCATGTC
ATCATCCCGTATGAAGGTCTGAGCGGCGACCAAATGGGCCAGATCGAAAAAATTTTTAAGG
TGGTGTACCCTGTGGATGATCATCACTTTAAGGTGATCCTGCACTATGGCACACTGGTAAT
CGACGGGGTTACGCCGAACATGATCGACTATTTCGGACGGCCGTATGAAGGCATCGCCGT
GTTCGACGGCAAAAAGATCACTGTAACAGGGACCCTGTGGAACGGCAACAAAATTATCGA
CGAGCGCCTGATCAACCCCGACGGCTCCCTGCTGTTCCGAGTAACCATCAACGGAGTGAC
CGGCTGGCGGCTGTGCGAACGCATTCTGGCGGACTACAAAGACCATGACGGTGATTATAA
AGATCATGACATCGATTACAAGGATGACGATGACAAGTAAGGCCGCGACTCTAGA

pcDNA3.1+/uORF nLuc (SacI/XbaI)
GAGCTCTCTGGCTAACTAGAGAACCCACTGCTTACTGGCTTATCGAAATTAATACGACTCA
CTATAGGcaacaacaacaacaacaacaacaacaacaacaacaacaacaacaacaacaacaacaacaacaacaacaac
aacaccatggtcaccatggtcaccatggtctaaacaacaacaacaacaaATGGTCTTCACACTCGAAGATTTCGT
TGGGGACTGGCGACAGACAGCCGGCTACAACCTGGACCAAGTCCTTGAACAGGGAGGTG
TGTCCAGTTTGTTTCAGAATCTCGGGGTGTCCGTAACTCCGATCCAAAGGATTGTCCTGAG
CGGTGAAAATGGGCTGAAGATCGACATCCATGTCATCATCCCGTATGAAGGTCTGAGCGG
CGACCAAATGGGCCAGATCGAAAAAATTTTTAAGGTGGTGTACCCTGTGGATGATCATCAC
TTTAAGGTGATCCTGCACTATGGCACACTGGTAATCGACGGGGTTACGCCGAACATGATCG
ACTATTTCGGACGGCCGTATGAAGGCATCGCCGTGTTCGACGGCAAAAAGATCACTGTAA
CAGGGACCCTGTGGAACGGCAACAAAATTATCGACGAGCGCCTGATCAACCCCGACGGCT
CCCTGCTGTTCCGAGTAACCATCAACGGAGTGACCGGCTGGCGGCTGTGCGAACGCATTC
TGGCGGACTACAAAGACCATGACGGTGATTATAAAGATCATGACATCGATTACAAGGATGA
CGATGACAAGTAAGGCCGCGACTCTAGA

pcDNA3.1+/mt-uORF nLuc (SacI/XbaI)
GAGCTCTCTGGCTAACTAGAGAACCCACTGCTTACTGGCTTATCGAAATTAATACGACTCA
CTATAGGcaacaacaacaacaacaacaacaacaacaacaacaacaacaacaacaacaacaacaacaacaacaacaac
aacaccaaagtcaccaaagtcaccaaagtctaaacaacaacaacaacaaATGGTCTTCACACTCGAAGATTTCG
TTGGGGACTGGCGACAGACAGCCGGCTACAACCTGGACCAAGTCCTTGAACAGGGAGGT
GTGTCCAGTTTGTTTCAGAATCTCGGGGTGTCCGTAACTCCGATCCAAAGGATTGTCCTGA
GCGGTGAAAATGGGCTGAAGATCGACATCCATGTCATCATCCCGTATGAAGGTCTGAGCG
GCGACCAAATGGGCCAGATCGAAAAAATTTTTAAGGTGGTGTACCCTGTGGATGATCATCA
CTTTAAGGTGATCCTGCACTATGGCACACTGGTAATCGACGGGGTTACGCCGAACATGATC
GACTATTTCGGACGGCCGTATGAAGGCATCGCCGTGTTCGACGGCAAAAAGATCACTGTA
ACAGGGACCCTGTGGAACGGCAACAAAATTATCGACGAGCGCCTGATCAACCCCGACGGC
TCCCTGCTGTTCCGAGTAACCATCAACGGAGTGACCGGCTGGCGGCTGTGCGAACGCATT

CTGGCGGACTACAAAGACCATGACGGTGATTATAAAGATCATGACATCGATTACAAGGATG
ACGATGACAAGTAAGGCCGCGACTCTAGA

pcDNA3.1+/10AUG uORF nLuc (SacI/XbaI)
GAGCTCTCTGGCTAACTAGAGAACCCACTGCTTACTGGCTTATCGAAATTAATACGACTCA
CTATAGGcaacaacaacaacaacaacaacaacaacaacaacaacaacaacaacaacaacaacaacaacaacaac
aacaccatgatgatgatgatgatgatgatgatgatgtaaacaacaacaacaacaaATGGTCTTCACACTCGAAGATT
TCGTTGGGGACTGGCGACAGACAGCCGGCTACAACCTGGACCAAGTCCTTGAACAGGGA
GGTGTGTCCAGTTTGTTTCAGAATCTCGGGGTGTCCGTAACTCCGATCCAAAGGATTGTCC
TGAGCGGTGAAAATGGGCTGAAGATCGACATCCATGTCATCATCCCGTATGAAGGTCTGA
GCGGCGACCAAATGGGCCAGATCGAAAAAATTTTTAAGGTGGTGTACCCTGTGGATGATCA
TCACTTTAAGGTGATCCTGCACTATGGCACACTGGTAATCGACGGGGTTACGCCGAACATG
ATCGACTATTTCGGACGGCCGTATGAAGGCATCGCCGTGTTCGACGGCAAAAAGATCACT
GTAACAGGGACCCTGTGGAACGGCAACAAAATTATCGACGAGCGCCTGATCAACCCCGAC
GGCTCCCTGCTGTTCCGAGTAACCATCAACGGAGTGACCGGCTGGCGGCTGTGCGAACG
CATTCTGGCGGACTACAAAGACCATGACGGTGATTATAAAGATCATGACATCGATTACAAG
GATGACGATGACAAGTAAGGCCGCGACTCTAGA

pcDNA3.1+/10AAA mt-uORF nLuc (SacI/XbaI)
GAGCTCTCTGGCTAACTAGAGAACCCACTGCTTACTGGCTTATCGAAATTAATACGACTCA
CTATAGGcaacaacaacaacaacaacaacaacaacaacaacaacaacaacaacaacaacaacaacaacaacaac
aacaccaaaaaaaaaaaaaaaaaaaaaaaaaaaaaataaacaacaacaacaacaaATGGTCTTCACACTCGAA
GATTTCGTTGGGGACTGGCGACAGACAGCCGGCTACAACCTGGACCAAGTCCTTGAACAG
GGAGGTGTGTCCAGTTTGTTTCAGAATCTCGGGGTGTCCGTAACTCCGATCCAAAGGATTG
TCCTGAGCGGTGAAAATGGGCTGAAGATCGACATCCATGTCATCATCCCGTATGAAGGTCT
GAGCGGCGACCAAATGGGCCAGATCGAAAAAATTTTTAAGGTGGTGTACCCTGTGGATGA
TCATCACTTTAAGGTGATCCTGCACTATGGCACACTGGTAATCGACGGGGTTACGCCGAAC
ATGATCGACTATTTCGGACGGCCGTATGAAGGCATCGCCGTGTTCGACGGCAAAAAGATC
ACTGTAACAGGGACCCTGTGGAACGGCAACAAAATTATCGACGAGCGCCTGATCAACCCC
GACGGCTCCCTGCTGTTCCGAGTAACCATCAACGGAGTGACCGGCTGGCGGCTGTGCGA
ACGCATTCTGGCGGACTACAAAGACCATGACGGTGATTATAAAGATCATGACATCGATTAC
AAGGATGACGATGACAAGTAAGGCCGCGACTCTAGA

pcDNA3.1+/large uORF nLuc (SacI/XbaI)
GAGCTCTCTGGCTAACTAGAGAACCCACTGCTTACTGGCTTATCGAAATTAATACGACTCA
CTATAGGcaacaacaacaacaacaacaacaacaacaacaacaacaacaacaacaacaacaacaacaacaacaac
aacaccatggtcaccatggtcaccatggtcGCAGAAATCGGTACTGGCTTTCCATTCGACCCCCATTATG
TGGAAGTCCTGGGCGAGCGCATGCACTACGTCGATGTTGGTCCGCGCGATGGCACCCCT
GTGCTGTTCCTGCACGGTAACCCGACCTCCTCCTACGTGTGGCGCAACATCATCCCGCAT
GTTGCACCGACCCATCGCTGCATTGCTCCAGACCTGATCGGTATGGGCAAATCCGACAAA
CCAGACCTGGGTTATTTCTTCGACGACCACGTCCGCTTCATGGATGCCTTCATCGAAGCCC
TGGGTCTGGAAGAGGTCGTCCTGGTCATTCACGACTGGGGCTCCGCTCTGGGTTTCCACT
GGGCCAAGCGCAATCCAGAGCGCGTCAAAGGTATTGCATTTATGGAGTTCATCCGCCCTA
TCCCGACCTGGGACGAATGGCCAGAATTTGCCCGCGAGACCTTCCAGGCCTTCCGCACCA
CCGACGTCGGCCGCAAGCTGATCATCGATCAGAACGTTTTTATCGAGGGTACGCTGCCGA
TGGGTGTCGTCCGCCCGCTGACTGAAGTCGAGATGGACCATTACCGCGAGCCGTTCCTGA
ATCCTGTTGACCGCGAGCCACTGTGGCGCTTCCCAAACGAGCTGCCAATCGCCGGTGAGC
CAGCGAACATCGTCGCGCTGGTCGAAGAATACATGGACTGGCTGCACCAGTCCCCTGTCC
CGAAGCTGCTGTTCTGGGGCACCCCAGGCGTTCTGATCCCACCGGCCGAAGCCGCTCGC
CTGGCCAAAAGCCTGCCTAACTGCAAGGCTGTGGACATCGGCCCGGGTCTGAATCTGCTG
CAAGAAGACAACCCGGACCTGATCGGCAGCGAGATCGCGCGCTGGCTGTCGACGCTCGA

GATTTCCGGCCACCGGTCGCAACCTTGGGTACCGCGGGCCCGGGATCCACCGGTCGCAA
CCTTGGTGAGCAAGGGCGAGGAGCTGTTCACCGGGGTGGTGCCCATCCTGGTCGAGCTG
GACGGCGACGTAAACGGCCACAAGTTCAGCGTGTCCGGCGAGGGCGAGGGCGATGCCAC
CTACGGCAAGCTGACCCTGAAGTTCATCTGCACCACCGGCAAGCTGCCCGTGCCCTGGCC
CACCCTCGTGACCACCCTGACCTACGGCGTGCAGTGCTTCAGCCGCTACCCCGACCACAT
GAAGCAGCACGACTTCTTCAAGTCCGCCATGCCCGAAGGCTACGTCCAGGAGCGCACCAT
CTTCTTCAAGGACGACGGCAACTACAAGACCCGCGCCGAGGTGAAGTTCGAGGGCGACA
CCCTGGTGAACCGCATCGAGCTGAAGGGCATCGACTTCAAGGAGGACGGCAACATCCTG
GGGCACAAGCTGGAGTACAACTACAACAGCCACAACGTCTATATCATGGCCGACAAGCAG
AAGAACGGCATCAAGGTGAACTTCAAGATCCGCCACAACATCGAGGACGGCAGCGTGCAG
CTCGCCGACCACTACCAGCAGAACACCCCCATCGGCGACGGCCCCGTGCTGCTGCCCGA
CAACCACTACCTGAGCACCCAGTCCGCCCTGAGCAAAGACCCCAACGAGAAGCGCGATCA
CATGGTCCTGCTGGAGTTCGTGACCGCCGCCGGGATCACTCTCGGCATGGACGAGCTGTA
CAAGtaaacaacaacaacaacaaATGGTCTTCACACTCGAAGATTTCGTTGGGGACTGGCGACAG
ACAGCCGGCTACAACCTGGACCAAGTCCTTGAACAGGGAGGTGTGTCCAGTTTGTTTCAG
AATCTCGGGGTGTCCGTAACTCCGATCCAAAGGATTGTCCTGAGCGGTGAAAATGGGCTG
AAGATCGACATCCATGTCATCATCCCGTATGAAGGTCTGAGCGGCGACCAAATGGGCCAG
ATCGAAAAAATTTTTAAGGTGGTGTACCCTGTGGATGATCATCACTTTAAGGTGATCCTGCA
CTATGGCACACTGGTAATCGACGGGGTTACGCCGAACATGATCGACTATTTCGGACGGCC
GTATGAAGGCATCGCCGTGTTCGACGGCAAAAAGATCACTGTAACAGGGACCCTGTGGAA
CGGCAACAAAATTATCGACGAGCGCCTGATCAACCCCGACGGCTCCCTGCTGTTCCGAGT
AACCATCAACGGAGTGACCGGCTGGCGGCTGTGCGAACGCATTCTGGCGGACTACAAAGA
CCATGACGGTGATTATAAAGATCATGACATCGATTACAAGGATGACGATGACAAGTAAGGC
CGCGACTCTAGA

pcDNA3.1+/uORF nLuc (SacI/XbaI)_115 nt spacer
GAGCTCTCTGGCTAACTAGAGAACCCACTGCTTACTGGCTTATCGAAATTAATACGACTCA
CTATAGGcaacaacaacaacaacaacaacaacaacaacaacaacaacaacaacaacaacaacaacaacaacaacaac
aacaccatggtcaccatggtcaccatggtctaaacaacaacaacaacaacaacaacaacaacaacaacaacaacaacaacaa
caacaacaacaacaacaacaacaacaacaacaacaacaacaacaacaacaacaacaacaacaacaacaaATGGTCTTCA
CACTCGAAGATTTCGTTGGGGACTGGCGACAGACAGCCGGCTACAACCTGGACCAAGTCC
TTGAACAGGGAGGTGTGTCCAGTTTGTTTCAGAATCTCGGGGTGTCCGTAACTCCGATCCA
AAGGATTGTCCTGAGCGGTGAAAATGGGCTGAAGATCGACATCCATGTCATCATCCCGTAT
GAAGGTCTGAGCGGCGACCAAATGGGCCAGATCGAAAAAATTTTTAAGGTGGTGTACCCT
GTGGATGATCATCACTTTAAGGTGATCCTGCACTATGGCACACTGGTAATCGACGGGGTTA
CGCCGAACATGATCGACTATTTCGGACGGCCGTATGAAGGCATCGCCGTGTTCGACGGCA
AAAAGATCACTGTAACAGGGACCCTGTGGAACGGCAACAAAATTATCGACGAGCGCCTGA
TCAACCCCGACGGCTCCCTGCTGTTCCGAGTAACCATCAACGGAGTGACCGGCTGGCGG
CTGTGCGAACGCATTCTGGCGGACTACAAAGACCATGACGGTGATTATAAAGATCATGACA
TCGATTACAAGGATGACGATGACAAGTAAGGCCGCGACTCTAGA

pcDNA3.1+/large uORF nLuc (SacI/XbaI)_115 nt spacer
GAGCTCTCTGGCTAACTAGAGAACCCACTGCTTACTGGCTTATCGAAATTAATACGACTCA
CTATAGGcaacaacaacaacaacaacaacaacaacaacaacaacaacaacaacaacaacaacaacaacaacaacaac
aacaccatggtcaccatggtcaccatggtcGCAGAAATCGGTACTGGCTTTCCATTCGACCCCCATTATG
TGGAAGTCCTGGGCGAGCGCATGCACTACGTCGATGTTGGTCCGCGCGATGGCACCCCT
GTGCTGTTCCTGCACGGTAACCCGACCTCCTCCTACGTGTGGCGCAACATCATCCCGCAT
GTTGCACCGACCCATCGCTGCATTGCTCCAGACCTGATCGGTATGGGCAAATCCGACAAA
CCAGACCTGGGTTATTTCTTCGACGACCACGTCCGCTTCATGGATGCCTTCATCGAAGCCC
TGGGTCTGGAAGAGGTCGTCCTGGTCATTCACGACTGGGGCTCCGCTCTGGGTTTCCACT
GGGCCAAGCGCAATCCAGAGCGCGTCAAAGGTATTGCATTTATGGAGTTCATCCGCCCTA

TCCCGACCTGGGACGAATGGCCAGAATTTGCCCGCGAGACCTTCCAGGCCTTCCGCACCA
CCGACGTCGGCCGCAAGCTGATCATCGATCAGAACGTTTTTATCGAGGGTACGCTGCCGA
TGGGTGTCGTCCGCCCGCTGACTGAAGTCGAGATGGACCATTACCGCGAGCCGTTCCTGA
ATCCTGTTGACCGCGAGCCACTGTGGCGCTTCCCAAACGAGCTGCCAATCGCCGGTGAGC
CAGCGAACATCGTCGCGCTGGTCGAAGAATACATGGACTGGCTGCACCAGTCCCCTGTCC
CGAAGCTGCTGTTCTGGGGCACCCCAGGCGTTCTGATCCCACCGGCCGAAGCCGCTCGC
CTGGCCAAAAGCCTGCCTAACTGCAAGGCTGTGGACATCGGCCCGGGTCTGAATCTGCTG
CAAGAAGACAACCCGGACCTGATCGGCAGCGAGATCGCGCGCTGGCTGTCGACGCTCGA
GATTTCCGGCCACCGGTCGCAACCTTGGGTACCGCGGGCCCGGGATCCACCGGTCGCAA
CCTTGGTGAGCAAGGGCGAGGAGCTGTTCACCGGGGTGGTGCCCATCCTGGTCGAGCTG
GACGGCGACGTAAACGGCCACAAGTTCAGCGTGTCCGGCGAGGGCGAGGGCGATGCCAC
CTACGGCAAGCTGACCCTGAAGTTCATCTGCACCACCGGCAAGCTGCCCGTGCCCTGGCC
CACCCTCGTGACCACCCTGACCTACGGCGTGCAGTGCTTCAGCCGCTACCCCGACCACAT
GAAGCAGCACGACTTCTTCAAGTCCGCCATGCCCGAAGGCTACGTCCAGGAGCGCACCAT
CTTCTTCAAGGACGACGGCAACTACAAGACCCGCGCCGAGGTGAAGTTCGAGGGCGACA
CCCTGGTGAACCGCATCGAGCTGAAGGGCATCGACTTCAAGGAGGACGGCAACATCCTG
GGGCACAAGCTGGAGTACAACTACAACAGCCACAACGTCTATATCATGGCCGACAAGCAG
AAGAACGGCATCAAGGTGAACTTCAAGATCCGCCACAACATCGAGGACGGCAGCGTGCAG
CTCGCCGACCACTACCAGCAGAACACCCCCATCGGCGACGGCCCCGTGCTGCTGCCCGA
CAACCACTACCTGAGCACCCAGTCCGCCCTGAGCAAAGACCCCAACGAGAAGCGCGATCA
CATGGTCCTGCTGGAGTTCGTGACCGCCGCCGGGATCACTCTCGGCATGGACGAGCTGTA
CAAGtaaacaacaacaacaacaacaacaacaacaacaacaacaacaacaacaacaacaacaacaacaacaac
aacaacaacaacaacaacaacaacaacaacaacaacaacaaATGGTCTTCACACTCGAAGATTTCGTT
GGGGACTGGCGACAGACAGCCGGCTACAACCTGGACCAAGTCCTTGAACAGGGAGGTGT
GTCCAGTTTGTTTCAGAATCTCGGGGTGTCCGTAACTCCGATCCAAAGGATTGTCCTGAGC
GGTGAAAATGGGCTGAAGATCGACATCCATGTCATCATCCCGTATGAAGGTCTGAGCGGC
GACCAAATGGGCCAGATCGAAAAAATTTTTAAGGTGGTGTACCCTGTGGATGATCATCACT
TTAAGGTGATCCTGCACTATGGCACACTGGTAATCGACGGGGTTACGCCGAACATGATCG
ACTATTTCGGACGGCCGTATGAAGGCATCGCCGTGTTCGACGGCAAAAAGATCACTGTAA
CAGGGACCCTGTGGAACGGCAACAAAATTATCGACGAGCGCCTGATCAACCCCGACGGCT
CCCTGCTGTTCCGAGTAACCATCAACGGAGTGACCGGCTGGCGGCTGTGCGAACGCATTC
TGGCGGACTACAAAGACCATGACGGTGATTATAAAGATCATGACATCGATTACAAGGATGA
CGATGACAAGTAAGGCCGCGACTCTAGA

pcDNA3.1+/truncated uORF nLuc (SacI/XbaI)

GAGCTCTCTGGCTAACTAGAGAACCCACTGCTTACTGGCTTATCGAAATTAATACGACTCA
CTATAGGcaacaacaacaacaacaacaacaacaacaacaacaacaacaacaacaacaacaacaacaacaacaac
aacaccatggtcaccatggtcaccatggtcGCAGAAATCGGTACTGGCTTTCCAGGCATGGACGAGCTG
TACAAGtaaacaacaacaacaacaaATGGTCTTCACACTCGAAGATTTCGTTGGGGACTGGCGAC
AGACAGCCGGCTACAACCTGGACCAAGTCCTTGAACAGGGAGGTGTGTCCAGTTTGTTTC
AGAATCTCGGGGTGTCCGTAACTCCGATCCAAAGGATTGTCCTGAGCGGTGAAAATGGGC
TGAAGATCGACATCCATGTCATCATCCCGTATGAAGGTCTGAGCGGCGACCAAATGGGCC
AGATCGAAAAAATTTTTAAGGTGGTGTACCCTGTGGATGATCATCACTTTAAGGTGATCCTG
CACTATGGCACACTGGTAATCGACGGGGTTACGCCGAACATGATCGACTATTTCGGACGG
CCGTATGAAGGCATCGCCGTGTTCGACGGCAAAAAGATCACTGTAACAGGGACCCTGTGG
AACGGCAACAAAATTATCGACGAGCGCCTGATCAACCCCGACGGCTCCCTGCTGTTCCGA
GTAACCATCAACGGAGTGACCGGCTGGCGGCTGTGCGAACGCATTCTGGCGGACTACAAA
GACCATGACGGTGATTATAAAGATCATGACATCGATTACAAGGATGACGATGACAAGTAAG
GCCGCGACTCTAGA

pcDNA3.1+/10AUG large uORF nLuc (SacI/XbaI)

GAGCTCTCTGGCTAACTAGAGAACCCACTGCTTACTGGCTTATCGAAATTAATACGACTCA
CTATAGGcaacaacaacaacaacaacaacaacaacaacaacaacaacaacaacaacaacaacaacaacaac
aacaccatgatgatgatgatgatgatgatgatgatgGCAGAAATCGGTACTGGCTTTCCATTCGACCCCCAT
TATGTGGAAGTCCTGGGCGAGCGCATGCACTACGTCGATGTTGGTCCGCGCGATGGCACC
CCTGTGCTGTTCCTGCACGGTAACCCGACCTCCTCCTACGTGTGGCGCAACATCATCCCG
CATGTTGCACCGACCCATCGCTGCATTGCTCCAGACCTGATCGGTATGGGCAAATCCGAC
AAACCAGACCTGGGTTATTTCTTCGACGACCACGTCCGCTTCATGGATGCCTTCATCGAAG
CCCTGGGTCTGGAAGAGGTCGTCCTGGTCATTCACGACTGGGGCTCCGCTCTGGGTTTCC
ACTGGGCCAAGCGCAATCCAGAGCGCGTCAAAGGTATTGCATTTATGGAGTTCATCCGCC
CTATCCCGACCTGGGACGAATGGCCAGAATTTGCCCGCGAGACCTTCCAGGCCTTCCGCA
CCACCGACGTCGGCCGCAAGCTGATCATCGATCAGAACGTTTTTATCGAGGGTACGCTGC
CGATGGGTGTCGTCCGCCCGCTGACTGAAGTCGAGATGGACCATTACCGCGAGCCGTTCC
TGAATCCTGTTGACCGCGAGCCACTGTGGCGCTTCCCAAACGAGCTGCCAATCGCCGGTG
AGCCAGCGAACATCGTCGCGCTGGTCGAAGAATACATGGACTGGCTGCACCAGTCCCCTG
TCCCGAAGCTGCTGTTCTGGGGCACCCCAGGCGTTCTGATCCCACCGGCCGAAGCCGCT
CGCCTGGCCAAAAGCCTGCCTAACTGCAAGGCTGTGGACATCGGCCCGGGTCTGAATCTG
CTGCAAGAAGACAACCCGGACCTGATCGGCAGCGAGATCGCGCGCTGGCTGTCGACGCT
CGAGATTTCCGGCCACCGGTCGCAACCTTGGGTACCGCGGCCCGGGATCCACCGGTCG
CAACCTTGGTGAGCAAGGGCGAGGAGCTGTTCACCGGGGTGGTGCCCATCCTGGTCGAG
CTGGACGGCGACGTAAACGGCCACAAGTTCAGCGTGTCCGGCGAGGGCGAGGGCGATGC
CACCTACGGCAAGCTGACCCTGAAGTTCATCTGCACCACCGGCAAGCTGCCCGTGCCCTG
GCCCACCCTCGTGACCACCCTGACCTACGGCGTGCAGTGCTTCAGCCGCTACCCCGACCA
CATGAAGCAGCACGACTTCTTCAAGTCCGCCATGCCCGAAGGCTACGTCCAGGAGCGCAC
CATCTTCTTCAAGGACGACGGCAACTACAAGACCCGCGCCGAGGTGAAGTTCGAGGGCGA
CACCCTGGTGAACCGCATCGAGCTGAAGGGCATCGACTTCAAGGAGGACGGCAACATCCT
GGGGCACAAGCTGGAGTACAACTACAACAGCCACAACGTCTATATCATGGCCGACAAGCA
GAAGAACGGCATCAAGGTGAACTTCAAGATCCGCCACAACATCGAGGACGGCAGCGTGCA
GCTCGCCGACCACTACCAGCAGAACACCCCCATCGGCGACGGCCCCGTGCTGCTGCCCG
ACAACCACTACCTGAGCACCCAGTCCGCCCTGAGCAAAGACCCCAACGAGAAGCGCGATC
ACATGGTCCTGCTGGAGTTCGTGACCGCCGCCGGGATCACTCTCGGCATGGACGAGCTGT
ACAAGtaaacaacaacaacaacaaATGGTCTTCACACTCGAAGATTTCGTTGGGGACTGGCGACA
GACAGCCGGCTACAACCTGGACCAAGTCCTTGAACAGGGAGGTGTGTCCAGTTTGTTTCA
GAATCTCGGGGTGTCCGTAACTCCGATCCAAAGGATTGTCCTGAGCGGTGAAAATGGGCT
GAAGATCGACATCCATGTCATCATCCCGTATGAAGGTCTGAGCGGCGACCAAATGGGCCA
GATCGAAAAAATTTTTAAGGTGGTGTACCCTGTGGATGATCATCACTTTAAGGTGATCCTGC
ACTATGGCACACTGGTAATCGACGGGGTTACGCCGAACATGATCGACTATTTCGGACGGC
CGTATGAAGGCATCGCCGTGTTCGACGGCAAAAAGATCACTGTAACAGGGACCCTGTGGA
ACGGCAACAAAATTATCGACGAGCGCCTGATCAACCCCGACGGCTCCCTGCTGTTCCGAG
TAACCATCAACGGAGTGACCGGCTGGCGGCTGTGCGAACGCATTCTGGCGGACTACAAAG
ACCATGACGGTGATTATAAAGATCATGACATCGATTACAAGGATGACGATGACAAGTAAGG
CCGCGACTCTAGA

pcDNA3.1+/10AUG truncated uORF nLuc (SacI/XbaI)
GAGCTCTCTGGCTAACTAGAGAACCCACTGCTTACTGGCTTATCGAAATTAATACGACTCA
CTATAGGcaacaacaacaacaacaacaacaacaacaacaacaacaacaacaacaacaacaacaacaacaac
aacaccatgatgatgatgatgatgatgatgatgatgGCAGAAATCGGTACTGGCTTTCCAGGCATGGACGAG
CTGTACAAGtaaacaacaacaacaacaaATGGTCTTCACACTCGAAGATTTCGTTGGGGACTGGC
GACAGACAGCCGGCTACAACCTGGACCAAGTCCTTGAACAGGGAGGTGTGTCCAGTTTGT
TTCAGAATCTCGGGGTGTCCGTAACTCCGATCCAAAGGATTGTCCTGAGCGGTGAAAATG
GGCTGAAGATCGACATCCATGTCATCATCCCGTATGAAGGTCTGAGCGGCGACCAAATGG
GCCAGATCGAAAAAATTTTTAAGGTGGTGTACCCTGTGGATGATCATCACTTTAAGGTGATC

CTGCACTATGGCACACTGGTAATCGACGGGGTTACGCCGAACATGATCGACTATTTCGGA
CGGCCGTATGAAGGCATCGCCGTGTTCGACGGCAAAAAGATCACTGTAACAGGGACCCTG
TGGAACGGCAACAAAATTATCGACGAGCGCCTGATCAACCCCGACGGCTCCCTGCTGTTC
CGAGTAACCATCAACGGAGTGACCGGCTGGCGGCTGTGCGAACGCATTCTGGCGGACTA
CAAAGACCATGACGGTGATTATAAAGATCATGACATCGATTACAAGGATGACGATGACAAG
TAAGGCCGCGACTCTAGA

pcDNA3.1+/large uORF (FFLuc) nLuc (SacI/XbaI)
GAGCTCTCTGGCTAACTAGAGAACCCACTGCTTACTGGCTTATCGAAATTAATACGACTCA
CTATAGGcaacaacaacaacaacaacaacaacaacaacaacaacaacaacaacaacaacaacaacaacaacaac
aacaccatggtcaccatggtcaccatggtcGAAGATGCCAAAAACATTAAGAAGGGCCCAGCGCCATTCT
ACCCACTCGAAGACGGGACCGCCGGCGAGCAGCTGCACAAAGCCATGAAGCGCTACGCC
CTGGTGCCCGGCACCATCGCCTTTACCGACGCACATATCGAGGTGGACATTACCTACGCC
GAGTACTTCGAGATGAGCGTTCGGCTGGCAGAAGCTATGAAGCGCTATGGGCTGAATACA
AACCATCGGATCGTGGTGTGCAGCGAGAATAGCTTGCAGTTCTTCATGCCCGTGTTGGGT
GCCCTGTTCATCGGTGTGGCTGTGGCCCCAGCTAACGACATCTACAACGAGCGCGAGCTG
CTGAACAGCATGGGCATCAGCCAGCCCACCGTCGTATTCGTGAGCAAGAAAGGGCTGCAA
AAGATCCTCAACGTGCAAAAGAAGCTACCGATCATACAAAAGATCATCATCATGGATAGCA
AGACCGACTACCAGGGCTTCCAAAGCATGTACACCTTCGTGACTTCCCATTTGCCACCCGG
CTTCAACGAGTACGACTTCGTGCCCGAGAGCTTCGACCGGGACAAAACCATCGCCCTGAT
CATGAACAGTAGTGGCAGTACCGGATTGCCCAAGGGCGTAGCCCTACCGCACCGCACCG
CTTGTGTCCGATTCAGTCATGCCCGCGACCCCATCTTCGGCAACCAGATCATCCCCGACA
CCGCTATCCTCAGCGTGGTGCCATTTCACCACGGCTTCGGCATGTTCACCACGCTGGGCT
ACTTGATCTGCGGCTTTCGGGTCGTGCTCATGTACCGCTTCGAGGAGGAGCTATTCTTGC
GCAGCTTGCAAGACTATAAGATTCAATCTGCCCTGCTGGTGCCCACACTATTTAGCTTCTTC
GCTAAGAGCACTCTCATCGACAAGTACGACCTAAGCAACTTGCACGAGATCGCCAGCGGC
GGGGCGCCGCTCAGCAAGGAGGTAGGTGAGGCCGTGGCCAAACGCTTCCACCTACCAGG
CATCCGCCAGGGCTACGGCCTGACAGAAACAACCAGCGCCATTCTGATCACCCCCGAAGG
GGACGACAAGCCTGGCGCAGTAGGCAAGGTGGTGCCCTTCTTCGAGGCTAAGGTGGTGG
ACTTGGACACCGGTAAGACACTGGGTGTGAACCAGCGCGGCGAGCTGTGCGTCCGTGGC
CCCATGATCATGAGCGGCTACGTTAACAACCCCGAGGCTACAAACGCTCTCATCGACAAG
GACGGCTGGCTGCACAGCGGCGACATCGCCTACTGGGACGAGGACGAGCACTTCTTCAT
CGTGGACCGGCTGAAGAGCCTGATCAAATACAAGGGCTACCAGGTAGCCCCAGCCGAACT
GGAGAGCATCCTGCTGCAACACCCCAACATCTTCGACGCCGGGGTCGCCGGCCTGCCCG
ACGACGATGCCGGCGAGCTGCCCGCCGCAGTCGTCGTGCTGGAACACGGTAAAACCATG
ACCGAGAAGGAGATCGTGGACTATGTGGCCAGCCAGGTTACAACCGCCAAGAAGCTGCG
CGGTGGTGTTGTGTTCGTGGACGAGGTGCCTAAAGGACTGACCGGCAAGTTGGACGCCC
GCAAGATCCGCGAGATTCTCATTAAGGCCAAGAAGGGCGGCAAGATCGCCGTGGGCAAAC
CGATTCCGAACCCGCTGCTGGGCCTGGATAGCACCtaaacaacaacaacaacaaATGGTCTTCAC
ACTCGAAGATTTCGTTGGGGACTGGCGACAGACAGCCGGCTACAACCTGGACCAAGTCCT
TGAACAGGGAGGTGTGTCCAGTTTGTTTCAGAATCTCGGGGTGTCCGTAACTCCGATCCAA
AGGATTGTCCTGAGCGGTGAAAATGGGCTGAAGATCGACATCCATGTCATCATCCCGTATG
AAGGTCTGAGCGGCGACCAAATGGGCCAGATCGAAAAAATTTTTAAGGTGGTGTACCCTGT
GGATGATCATCACTTTAAGGTGATCCTGCACTATGGCACACTGGTAATCGACGGGGTTACG
CCGAACATGATCGACTATTTCGGACGGCCGTATGAAGGCATCGCCGTGTTCGACGGCAAA
AAGATCACTGTAACAGGGACCCTGTGGAACGGCAACAAAATTATCGACGAGCGCCTGATC
AACCCCGACGGCTCCCTGCTGTTCCGAGTAACCATCAACGGAGTGACCGGCTGGCGGCT
GTGCGAACGCATTCTGGCGGACTACAAAGACCATGACGGTGATTATAAAGATCATGACATC
GATTACAAGGATGACGATGACAAGTAAGGCCGCGACTCTAGA

pcDNA3.1+/truncated uORF (FFluc) nLuc (SacI/XbaI)

GAGCTCTCTGGCTAACTAGAGAACCCACTGCTTACTGGCTTATCGAAATTAATACGACTCA
CTATAGGcaacaacaacaacaacaacaacaacaacaacaacaacaacaacaacaacaacaacaacaacaacaacaac
aacaccatggtcaccatggtcaccatggtcGAAGATGCCAAAAACATTAAGAAGCTGCTGGGCCTGGATA
GCACCtaaacaacaacaacaacaaATGGTCTTCACACTCGAAGATTTCGTTGGGGACTGGCGACA
GACAGCCGGCTACAACCTGGACCAAGTCCTTGAACAGGGAGGTGTGTCCAGTTTGTTTCA
GAATCTCGGGGTGTCCGTAACTCCGATCCAAAGGATTGTCCTGAGCGGTGAAAATGGGCT
GAAGATCGACATCCATGTCATCATCCCGTATGAAGGTCTGAGCGGCGACCAAATGGGCCA
GATCGAAAAAATTTTTAAGGTGGTGTACCCTGTGGATGATCATCACTTTAAGGTGATCCTGC
ACTATGGCACACTGGTAATCGACGGGGTTACGCCGAACATGATCGACTATTTCGGACGGC
CGTATGAAGGCATCGCCGTGTTCGACGGCAAAAAGATCACTGTAACAGGGACCCTGTGGA
ACGGCAACAAAATTATCGACGAGCGCCTGATCAACCCCGACGGCTCCCTGCTGTTCCGAG
TAACCATCAACGGAGTGACCGGCTGGCGGCTGTGCGAACGCATTCTGGCGGACTACAAAG
ACCATGACGGTGATTATAAAGATCATGACATCGATTACAAGGATGACGATGACAAGTAAGG
CCGCGACTCTAGA

pcDNA3.1+/10AUG large uORF (FFLuc) nLuc (SacI/XbaI)
GAGCTCTCTGGCTAACTAGAGAACCCACTGCTTACTGGCTTATCGAAATTAATACGACTCA
CTATAGGcaacaacaacaacaacaacaacaacaacaacaacaacaacaacaacaacaacaacaacaacaacaacaac
aacaccatgatgatgatgatgatgatgatgatgatgGAAGATGCCAAAAACATTAAGAAGGGCCCAGCGCCA
TTCTACCCACTCGAAGACGGGACCGCCGGCGAGCAGCTGCACAAAGCCATGAAGCGCTA
CGCCCTGGTGCCCGGCACCATCGCCTTTACCGACGCACATATCGAGGTGGACATTACCTA
CGCCGAGTACTTCGAGATGAGCGTTCGGCTGGCAGAAGCTATGAAGCGCTATGGGCTGAA
TACAAACCATCGGATCGTGGTGTGCAGCGAGAATAGCTTGCAGTTCTTCATGCCCGTGTTG
GGTGCCCTGTTCATCGGTGTGGCTGTGGCCCCAGCTAACGACATCTACAACGAGCGCGAG
CTGCTGAACAGCATGGGCATCAGCCAGCCCACCGTCGTATTCGTGAGCAAGAAAGGGCTG
CAAAAGATCCTCAACGTGCAAAAGAAGCTACCGATCATACAAAAGATCATCATCATGGATA
GCAAGACCGACTACCAGGGCTTCCAAAGCATGTACACCTTCGTGACTTCCCATTTGCCACC
CGGCTTCAACGAGTACGACTTCGTGCCCGAGAGCTTCGACCGGGACAAAACCATCGCCCT
GATCATGAACAGTAGTGGCAGTACCGGATTGCCCAAGGGCGTAGCCCTACCGCACCGCAC
CGCTTGTGTCCGATTCAGTCATGCCCGCGACCCCATCTTCGGCAACCAGATCATCCCCGA
CACCGCTATCCTCAGCGTGGTGCCATTTCACCACGGCTTCGGCATGTTCACCACGCTGGG
CTACTTGATCTGCGGCTTTCGGGTCGTGCTCATGTACCGCTTCGAGGAGGAGCTATTCTTG
CGCAGCTTGCAAGACTATAAGATTCAATCTGCCCTGCTGGTGCCCACACTATTTAGCTTCTT
CGCTAAGAGCACTCTCATCGACAAGTACGACCTAAGCAACTTGCACGAGATCGCCAGCGG
CGGGGCGCCGCTCAGCAAGGAGGTAGGTGAGGCCGTGGCCAAACGCTTCCACCTACCAG
GCATCCGCCAGGGCTACGGCCTGACAGAAACAACCAGCGCCATTCTGATCACCCCCGAAG
GGGACGACAAGCCTGGCGCAGTAGGCAAGGTGGTGCCCTTCTTCGAGGCTAAGGTGGTG
GACTTGGACACCGGTAAGACACTGGGTGTGAACCAGCGCGGCGAGCTGTGCGTCCGTGG
CCCCATGATCATGAGCGGCTACGTTAACAACCCCGAGGCTACAAACGCTCTCATCGACAA
GGACGGCTGGCTGCACAGCGGCGACATCGCCTACTGGGACGAGGACGAGCACTTCTTCA
TCGTGGACCGGCTGAAGAGCCTGATCAAATACAAGGGCTACCAGGTAGCCCCAGCCGAAC
TGGAGAGCATCCTGCTGCAACACCCCAACATCTTCGACGCCGGGGTCGCCGGCCTGCCC
GACGACGATGCCGGCGAGCTGCCCGCCGCAGTCGTCGTGCTGGAACACGGTAAAACCAT
GACCGAGAAGGAGATCGTGGACTATGTGGCCAGCCAGGTTACAACCGCCAAGAAGCTGC
GCGGTGGTGTTGTGTTCGTGGACGAGGTGCCTAAAGGACTGACCGGCAAGTTGGACGCC
CGCAAGATCCGCGAGATTCTCATTAAGGCCAAGAAGGGCGGCAAGATCGCCGTGGGCAAA
CCGATTCCGAACCCGCTGCTGGGCCTGGATAGCACCtaaacaacaacaacaacaaATGGTCTTCA
CACTCGAAGATTTCGTTGGGGACTGGCGACAGACAGCCGGCTACAACCTGGACCAAGTCC
TTGAACAGGGAGGTGTGTCCAGTTTGTTTCAGAATCTCGGGGTGTCCGTAACTCCGATCCA
AAGGATTGTCCTGAGCGGTGAAAATGGGCTGAAGATCGACATCCATGTCATCATCCCGTAT
GAAGGTCTGAGCGGCGACCAAATGGGCCAGATCGAAAAAATTTTTAAGGTGGTGTACCCT

GTGGATGATCATCACTTTAAGGTGATCCTGCACTATGGCACACTGGTAATCGACGGGGTTA
CGCCGAACATGATCGACTATTTCGGACGGCCGTATGAAGGCATCGCCGTGTTCGACGGCA
AAAAGATCACTGTAACAGGGACCCTGTGGAACGGCAACAAAATTATCGACGAGCGCCTGA
TCAACCCCGACGGCTCCCTGCTGTTCCGAGTAACCATCAACGGAGTGACCGGCTGGCGG
CTGTGCGAACGCATTCTGGCGGACTACAAAGACCATGACGGTGATTATAAAGATCATGACA
TCGATTACAAGGATGACGATGACAAGTAAGGCCGCGACTCTAGA

pcDNA3.1+/overlapping uORF nLuc (SacI/XbaI)
GAGCTCTCTGGCTAACTAGAGAACCCACTGCTTACTGGCTTATCGAAATTAATACGACTCA
CTATAGGcaacaacaacaacaacaacaacaacaacaacaacaacaacaacaacaacaacaacaacaacaacaacaacaacaacaac
aacaccatggtcaccatggtcaccatggtcGTGAGCAAGGGCGAGGATGGTCTTCACACTCGAAGATTTC
GTTGGGGACTGGCGACAGACAGCCGGCTACAACCTGGACCAAGTCCTTGAACAGGGAGG
TGTGTCCAGTTTGTTTCAGAATCTCGGGGTGTCCGTAACTCCGATCCAAAGGATTGTCCTG
AGCGGTGAAAATGGGCTGAAGATCGACATCCATGTCATCATCCCGTATGAAGGTCTGAGC
GGCGACCAAATGGGCCAGATCGAAAAAATTTTTAAGGTGGTGTACCCTGTGGATGATCATC
ACTTTAAGGTGATCCTGCACTATGGCACACTGGTAATCGACGGGGTTACGCCGAACATGAT
CGACTATTTCGGACGGCCGTATGAAGGCATCGCCGTGTTCGACGGCAAAAAGATCACTGT
AACAGGGACCCTGTGGAACGGCAACAAAATTATCGACGAGCGCCTGATCAACCCCGACGG
CTCCCTGCTGTTCCGAGTAACCATCAACGGAGTGACCGGCTGGCGGCTGTGCGAACGCAT
TCTGGCGGACTACAAAGACCATGACGGTGATTATAAAGATCATGACATCGATTACAAGGAT
GACGATGACAAGTAAGGCCGCGACTCTAGA

pcDNA3.1+/mt-overlapping uORF nLuc (SacI/XbaI)
GAGCTCTCTGGCTAACTAGAGAACCCACTGCTTACTGGCTTATCGAAATTAATACGACTCA
CTATAGGcaacaacaacaacaacaacaacaacaacaacaacaacaacaacaacaacaacaacaacaacaacaacaacaacaacaac
aacaccaaagtcaccaaagtcaccaaagtcGTGAGCAAGGGCGAGGATGGTCTTCACACTCGAAGATT
TCGTTGGGGACTGGCGACAGACAGCCGGCTACAACCTGGACCAAGTCCTTGAACAGGGA
GGTGTGTCCAGTTTGTTTCAGAATCTCGGGGTGTCCGTAACTCCGATCCAAAGGATTGTCC
TGAGCGGTGAAAATGGGCTGAAGATCGACATCCATGTCATCATCCCGTATGAAGGTCTGA
GCGGCGACCAAATGGGCCAGATCGAAAAAATTTTTAAGGTGGTGTACCCTGTGGATGATCA
TCACTTTAAGGTGATCCTGCACTATGGCACACTGGTAATCGACGGGGTTACGCCGAACATG
ATCGACTATTTCGGACGGCCGTATGAAGGCATCGCCGTGTTCGACGGCAAAAAGATCACT
GTAACAGGGACCCTGTGGAACGGCAACAAAATTATCGACGAGCGCCTGATCAACCCCGAC
GGCTCCCTGCTGTTCCGAGTAACCATCAACGGAGTGACCGGCTGGCGGCTGTGCGAACG
CATTCTGGCGGACTACAAAGACCATGACGGTGATTATAAAGATCATGACATCGATTACAAG
GATGACGATGACAAGTAAGGCCGCGACTCTAGA

pcDNA3.1+/10AUG truncated uORF (FFLuc) nLuc (SacI/XbaI)
GAGCTCTCTGGCTAACTAGAGAACCCACTGCTTACTGGCTTATCGAAATTAATACGACTCA
CTATAGGcaacaacaacaacaacaacaacaacaacaacaacaacaacaacaacaacaacaacaacaacaacaacaacaacaacaac
aacaccatgatgatgatgatgatgatgatgatgatgGAAGATGCCAAAAACATTAAGAAGCTGCTGGGCCTG
GATAGCACCtaaacaacaacaacaacaaATGGTCTTCACACTCGAAGATTTCGTTGGGGACTGGC
GACAGACAGCCGGCTACAACCTGGACCAAGTCCTTGAACAGGGAGGTGTGTCCAGTTTGT
TTCAGAATCTCGGGGTGTCCGTAACTCCGATCCAAAGGATTGTCCTGAGCGGTGAAAATG
GGCTGAAGATCGACATCCATGTCATCATCCCGTATGAAGGTCTGAGCGGCGACCAAATGG
GCCAGATCGAAAAAATTTTTAAGGTGGTGTACCCTGTGGATGATCATCACTTTAAGGTGATC
CTGCACTATGGCACACTGGTAATCGACGGGGTTACGCCGAACATGATCGACTATTTCGGA
CGGCCGTATGAAGGCATCGCCGTGTTCGACGGCAAAAAGATCACTGTAACAGGGACCCTG
TGGAACGGCAACAAAATTATCGACGAGCGCCTGATCAACCCCGACGGCTCCCTGCTGTTC
CGAGTAACCATCAACGGAGTGACCGGCTGGCGGCTGTGCGAACGCATTCTGGCGGACTA

CAAAGACCATGACGGTGATTATAAAGATCATGACATCGATTACAAGGATGACGATGACAAG
TAAGGCCGCGACTCTAGA

pcDNA3.1+/uORF-P2A-nLuc (SacI/XbaI)
GAGCTCTCTGGCTAACTAGAGAACCCACTGCTTACTGGCTTATCGAAATTAATACGACTCA
CTATAGGcaacaacaacaacaacaacaacaacaacaacaacaacaacaacaacaacaacaacaacaacaac
aacaccatggtcaccatggtcaccatggtcGGAAGCGGAGCTACTAACTTCAGCCTGCTGAAGCAGGCT
GGAGACGTGGAGGAGAACCCTGGACCTATGGTCTTCACACTCGAAGATTTCGTTGGGGAC
TGGCGACAGACAGCCGGCTACAACCTGGACCAAGTCCTTGAACAGGGAGGTGTGTCCAGT
TTGTTTCAGAATCTCGGGGTGTCCGTAACTCCGATCCAAAGGATTGTCCTGAGCGGTGAAA
ATGGGCTGAAGATCGACATCCATGTCATCATCCCGTATGAAGGTCTGAGCGGCGACCAAA
TGGGCCAGATCGAAAAAATTTTTAAGGTGGTGTACCCTGTGGATGATCATCACTTTAAGGT
GATCCTGCACTATGGCACACTGGTAATCGACGGGGTTACGCCGAACATGATCGACTATTTC
GGACGGCCGTATGAAGGCATCGCCGTGTTCGACGGCAAAAAGATCACTGTAACAGGGACC
CTGTGGAACGGCAACAAAATTATCGACGAGCGCCTGATCAACCCCGACGGCTCCCTGCTG
TTCCGAGTAACCATCAACGGAGTGACCGGCTGGCGGCTGTGCGAACGCATTCTGGCGGA
CTACAAAGACCATGACGGTGATTATAAAGATCATGACATCGATTACAAGGATGACGATGAC
AAGTAAGGCCGCGACTCTAGA

pcDNA3.1+/large uORF-P2A-nLuc (SacI/XbaI)
GAGCTCTCTGGCTAACTAGAGAACCCACTGCTTACTGGCTTATCGAAATTAATACGACTCA
CTATAGGcaacaacaacaacaacaacaacaacaacaacaacaacaacaacaacaacaacaacaacaacaac
aacaccatggtcaccatggtcaccatggtcGCAGAAATCGGTACTGGCTTTCCATTCGACCCCCATTATG
TGGAAGTCCTGGGCGAGCGCATGCACTACGTCGATGTTGGTCCGCGCGATGGCACCCCT
GTGCTGTTCCTGCACGGTAACCCGACCTCCTCCTACGTGTGGCGCAACATCATCCCGCAT
GTTGCACCGACCCATCGCTGCATTGCTCCAGACCTGATCGGTATGGGCAAATCCGACAAA
CCAGACCTGGGTTATTTCTTCGACGACCACGTCCGCTTCATGGATGCCTTCATCGAAGCCC
TGGGTCTGGAAGAGGTCGTCCTGGTCATTCACGACTGGGGCTCCGCTCTGGGTTTCCACT
GGGCCAAGCGCAATCCAGAGCGCGTCAAAGGTATTGCATTTATGGAGTTCATCCGCCCTA
TCCCGACCTGGGACGAATGGCCAGAATTTGCCCGCGAGACCTTCCAGGCCTTCCGCACCA
CCGACGTCGGCCGCAAGCTGATCATCGATCAGAACGTTTTTATCGAGGGTACGCTGCCGA
TGGGTGTCGTCCGCCCGCTGACTGAAGTCGAGATGGACCATTACCGCGAGCCGTTCCTGA
ATCCTGTTGACCGCGAGCCACTGTGGCGCTTCCCAAACGAGCTGCCAATCGCCGGTGAGC
CAGCGAACATCGTCGCGCTGGTCGAAGAATACATGGACTGGCTGCACCAGTCCCCTGTCC
CGAAGCTGCTGTTCTGGGGCACCCCAGGCGTTCTGATCCCACCGGCCGAAGCCGCTCGC
CTGGCCAAAAGCCTGCCTAACTGCAAGGCTGTGGACATCGGCCCGGGTCTGAATCTGCTG
CAAGAAGACAACCCGGACCTGATCGGCAGCGAGATCGCGCGCTGGCTGTCGACGCTCGA
GATTTCCGGCCACCGGTCGCAACCTTGGGTACCGCGGGCCCGGGATCCACCGGTCGCAA
CCTTGGTGAGCAAGGGCGAGGAGCTGTTCACCGGGGTGGTGCCCATCCTGGTCGAGCTG
GACGGCGACGTAAACGGCCACAAGTTCAGCGTGTCCGGCGAGGGCGAGGGCGATGCCAC
CTACGGCAAGCTGACCCTGAAGTTCATCTGCACCACCGGCAAGCTGCCCGTGCCCTGGCC
CACCCTCGTGACCACCCTGACCTACGGCGTGCAGTGCTTCAGCCGCTACCCCGACCACAT
GAAGCAGCACGACTTCTTCAAGTCCGCCATGCCCGAAGGCTACGTCCAGGAGCGCACCAT
CTTCTTCAAGGACGACGGCAACTACAAGACCCGCGCCGAGGTGAAGTTCGAGGGCGACA
CCCTGGTGAACCGCATCGAGCTGAAGGGCATCGACTTCAAGGAGGACGGCAACATCCTG
GGGCACAAGCTGGAGTACAACTACAACAGCCACAACGTCTATATCATGGCCGACAAGCAG
AAGAACGGCATCAAGGTGAACTTCAAGATCCGCCACAACATCGAGGACGGCAGCGTGCAG
CTCGCCGACCACTACCAGCAGAACACCCCCATCGGCGACGGCCCCGTGCTGCTGCCCGA
CAACCACTACCTGAGCACCCAGTCCGCCCTGAGCAAAGACCCCAACGAGAAGCGCGATCA
CATGGTCCTGCTGGAGTTCGTGACCGCCGCCGGGATCACTCTCGGCATGGACGAGCTGTA
CAAGGGAAGCGGAGCTACTAACTTCAGCCTGCTGAAGCAGGCTGGAGACGTGGAGGAGA

ACCCTGGACCTATGGTCTTCACACTCGAAGATTTCGTTGGGGACTGGCGACAGACAGCCG
GCTACAACCTGGACCAAGTCCTTGAACAGGGAGGTGTGTCCAGTTTGTTTCAGAATCTCGG
GGTGTCCGTAACTCCGATCCAAAGGATTGTCCTGAGCGGTGAAAATGGGCTGAAGATCGA
CATCCATGTCATCATCCCGTATGAAGGTCTGAGCGGCGACCAAATGGGCCAGATCGAAAA
AATTTTTAAGGTGGTGTACCCTGTGGATGATCATCACTTTAAGGTGATCCTGCACTATGGCA
CACTGGTAATCGACGGGGTTACGCCGAACATGATCGACTATTTCGGACGGCCGTATGAAG
GCATCGCCGTGTTCGACGGCAAAAAGATCACTGTAACAGGGACCCTGTGGAACGGCAACA
AAATTATCGACGAGCGCCTGATCAACCCCGACGGCTCCCTGCTGTTCCGAGTAACCATCAA
CGGAGTGACCGGCTGGCGGCTGTGCGAACGCATTCTGGCGGACTACAAAGACCATGACG
GTGATTATAAAGATCATGACATCGATTACAAGGATGACGATGACAAGTAAGGCCGCGACTC
TAGA

pcDNA3.1+/PV IRES no uORF nLuc (SacI/XbaI)
GAGCTCTCTGGCTAACTAGAGAACCCACTGCTTACTGGCTTATCGAAATTAATACGACTCA
CTATAGGGCTCGAG**TTGGGGCGCGTGGTGGCGGCTGCAGCCGCCACCACGCGCCCCGG**
aaaaaAAGCTTGGGCTGCAGGTCTTAAAACAGCTCTGGGGTTGTACCCACCCCAGAGGCCC
ACGTGGCGGCTAGTACTCCGGTATTGCGGTACCTTTGTACGCCTGTTTTATACTCCCTTCC
CCCGTAACTTAGAAGCACAATGTCCAAGTTCAATAGGAGGGGGTACAAACCAGTACCACCA
CGAACAAGCACTTCTGTTCCCCCGGTGAGGCTGTATAGGCTGTTTCCACGGCTAAAAGCG
GCTGATCCGTTATCCGCTCATGTACTTCGAGAAGCCTAGTATCACCTTGGAATCTTCGATG
CGTTGCGCTCAACACTCAACCCCAGAGTGTAGCTTAGGTCGATGAGTCTGGACGTTCCTCA
CCGGCGACGGTGGTCCAGGCTGCGTTGGCGGCCTACCTGTGGCCCAAAGCCACAGGACG
CTAGTTGTGAACAAGGTGTGAAGAGCCTATTGAGCTACCTGAGAGTCCTCCGGCCCCTGA
ATGCGGCTAATCCTAACCACGGAGCAGGCAGTGGCAATCCAGCGACCAGCCTGTCGTAAC
GCGCAAGTTCGTGGCGGAACCGACTACTTTGGGTGTCCGTGTTTCCTTTTATTTTTACAAT
GGCTGCTTATGGTGACAATCATTGATTGTTATCATAAAGCAAATTGGATTGGCCATCCGGT
GAGAATTTGATTATTAAATTACTCTCTTGTTGGGATTGCTCCTTTGAAATCTTGTGCACTCAC
ACCTATTGGAATTACCTCATTGTTAAGATAcaacaacaacaacaacaacaacaacaacaacaacaaca
acaacaacaacaacaacaacaacaacaacaaATGGTCTTCACACTCGAAGATTTCGTTGGGGACTGGC
GACAGACAGCCGGCTACAACCTGGACCAAGTCCTTGAACAGGGAGGTGTGTCCAGTTTGT
TTCAGAATCTCGGGGTGTCCGTAACTCCGATCCAAAGGATTGTCCTGAGCGGTGAAAATG
GGCTGAAGATCGACATCCATGTCATCATCCCGTATGAAGGTCTGAGCGGCGACCAAATGG
GCCAGATCGAAAAAATTTTTAAGGTGGTGTACCCTGTGGATGATCATCACTTTAAGGTGATC
CTGCACTATGGCACACTGGTAATCGACGGGGTTACGCCGAACATGATCGACTATTTCGGA
CGGCCGTATGAAGGCATCGCCGTGTTCGACGGCAAAAAGATCACTGTAACAGGGACCCTG
TGGAACGGCAACAAAATTATCGACGAGCGCCTGATCAACCCCGACGGCTCCCTGCTGTTC
CGAGTAACCATCAACGGAGTGACCGGCTGGCGGCTGTGCGAACGCATTCTGGCGGACTA
CAAAGACCATGACGGTGATTATAAAGATCATGACATCGATTACAAGGATGACGATGACAAG
TAAGGCCGCGACTCTAGA

pcDNA3.1+/PV IRES uORF nLuc (SacI/XbaI)_16 nt spacer
GAGCTCTCTGGCTAACTAGAGAACCCACTGCTTACTGGCTTATCGAAATTAATACGACTCA
CTATAGGGCTCGAG**TTGGGGCGCGTGGTGGCGGCTGCAGCCGCCACCACGCGCCCCGG**
aaaaaAAGCTTGGGCTGCAGGTCTTAAAACAGCTCTGGGGTTGTACCCACCCCAGAGGCCC
ACGTGGCGGCTAGTACTCCGGTATTGCGGTACCTTTGTACGCCTGTTTTATACTCCCTTCC
CCCGTAACTTAGAAGCACAATGTCCAAGTTCAATAGGAGGGGGTACAAACCAGTACCACCA
CGAACAAGCACTTCTGTTCCCCCGGTGAGGCTGTATAGGCTGTTTCCACGGCTAAAAGCG
GCTGATCCGTTATCCGCTCATGTACTTCGAGAAGCCTAGTATCACCTTGGAATCTTCGATG
CGTTGCGCTCAACACTCAACCCCAGAGTGTAGCTTAGGTCGATGAGTCTGGACGTTCCTCA
CCGGCGACGGTGGTCCAGGCTGCGTTGGCGGCCTACCTGTGGCCCAAAGCCACAGGACG
CTAGTTGTGAACAAGGTGTGAAGAGCCTATTGAGCTACCTGAGAGTCCTCCGGCCCCTGA

ATGCGGCTAATCCTAACCACGGAGCAGGCAGTGGCAATCCAGCGACCAGCCTGTCGTAAC
GCGCAAGTTCGTGGCGGAACCGACTACTTTGGGTGTCCGTGTTTCCTTTTATTTTTACAAT
GGCTGCTTATGGTGACAATCATTGATTGTTATCATAAAGCAAATTGGATTGGCCATCCGGT
GAGAATTTGATTATTAAATTACTCTCTTGTTGGGATTGCTCCTTTGAAATCTTGTGCACTCAC
ACCTATTGGAATTACCTCATTGTTAAGATAcaacaacaacaacaacaacaacaacaacaacaacaaca
acaacaacaacaacaacaacaacaacaacaacaccatggtcaccatggtcaccatggtctaaacaacaacaacaacaaATG
GTCTTCACACTCGAAGATTTCGTTGGGGACTGGCGACAGACAGCCGGCTACAACCTGGAC
CAAGTCCTTGAACAGGGAGGTGTGTCCAGTTTGTTTCAGAATCTCGGGGTGTCCGTAACTC
CGATCCAAAGGATTGTCCTGAGCGGTGAAAATGGGCTGAAGATCGACATCCATGTCATCAT
CCCGTATGAAGGTCTGAGCGGCGACCAAATGGGCCAGATCGAAAAAATTTTTAAGGTGGT
GTACCCTGTGGATGATCATCACTTTAAGGTGATCCTGCACTATGGCACACTGGTAATCGAC
GGGGTTACGCCGAACATGATCGACTATTTCGGACGGCCGTATGAAGGCATCGCCGTGTTC
GACGGCAAAAAGATCACTGTAACAGGGACCCTGTGGAACGGCAACAAAATTATCGACGAG
CGCCTGATCAACCCCGACGGCTCCCTGCTGTTCCGAGTAACCATCAACGGAGTGACCGGC
TGGCGGCTGTGCGAACGCATTCTGGCGGACTACAAAGACCATGACGGTGATTATAAAGAT
CATGACATCGATTACAAGGATGACGATGACAAGTAAGGCCGCGACTCTAGA

pcDNA3.1+/PV IRES uORF nLuc (SacI/XbaI)_115 nt spacer
GAGCTCTCTGGCTAACTAGAGAACCCACTGCTTACTGGCTTATCGAAATTAATACGACTCA
CTATAGGGCTCGAG**TTGGGGCGCGTGGTGGCGGCTGCAGCCGCCACCACGCGCCCCGG**
aaaaaAAGCTTGGGCTGCAGGTCTTAAAACAGCTCTGGGGTTGTACCCACCCCAGAGGCCC
ACGTGGCGGCTAGTACTCCGGTATTGCGGTACCTTTGTACGCCTGTTTTATACTCCCTTCC
CCCGTAACTTAGAAGCACAATGTCCAAGTTCAATAGGAGGGGGTACAAACCAGTACCACCA
CGAACAAGCACTTCTGTTCCCCCGGTGAGGCTGTATAGGCTGTTTCCACGGCTAAAAGCG
GCTGATCCGTTATCCGCTCATGTACTTCGAGAAGCCTAGTATCACCTTGGAATCTTCGATG
CGTTGCGCTCAACACTCAACCCCAGAGTGTAGCTTAGGTCGATGAGTCTGGACGTTCCTCA
CCGGCGACGGTGGTCCAGGCTGCGTTGGCGGCCTACCTGTGGCCCAAAGCCACAGGACG
CTAGTTGTGAACAAGGTGTGAAGAGCCTATTGAGCTACCTGAGAGTCCTCCGGCCCCTGA
ATGCGGCTAATCCTAACCACGGAGCAGGCAGTGGCAATCCAGCGACCAGCCTGTCGTAAC
GCGCAAGTTCGTGGCGGAACCGACTACTTTGGGTGTCCGTGTTTCCTTTTATTTTTACAAT
GGCTGCTTATGGTGACAATCATTGATTGTTATCATAAAGCAAATTGGATTGGCCATCCGGT
GAGAATTTGATTATTAAATTACTCTCTTGTTGGGATTGCTCCTTTGAAATCTTGTGCACTCAC
ACCTATTGGAATTACCTCATTGTTAAGATAcaacaacaacaacaacaacaacaacaacaacaacaaca
acaacaacaacaacaacaacaacaacaacaacaccatggtcaccatggtcaccatggtctaaacaacaacaacaacaacaac
aacaacaacaacaacaacaacaacaacaacaacaacaacaacaacaacaacaacaacaacaacaacaacaacaacaacaaca
acaacaacaacaacaaATGGTCTTCACACTCGAAGATTTCGTTGGGGACTGGCGACAGACAGCC
GGCTACAACCTGGACCAAGTCCTTGAACAGGGAGGTGTGTCCAGTTTGTTTCAGAATCTCG
GGGTGTCCGTAACTCCGATCCAAAGGATTGTCCTGAGCGGTGAAAATGGGCTGAAGATCG
ACATCCATGTCATCATCCCGTATGAAGGTCTGAGCGGCGACCAAATGGGCCAGATCGAAA
AAATTTTTAAGGTGGTGTACCCTGTGGATGATCATCACTTTAAGGTGATCCTGCACTATGGC
ACACTGGTAATCGACGGGGTTACGCCGAACATGATCGACTATTTCGGACGGCCGTATGAA
GGCATCGCCGTGTTCGACGGCAAAAAGATCACTGTAACAGGGACCCTGTGGAACGGCAAC
AAAATTATCGACGAGCGCCTGATCAACCCCGACGGCTCCCTGCTGTTCCGAGTAACCATCA
ACGGAGTGACCGGCTGGCGGCTGTGCGAACGCATTCTGGCGGACTACAAAGACCATGAC
GGTGATTATAAAGATCATGACATCGATTACAAGGATGACGATGACAAGTAAGGCCGCGACT
CTAGA

pcDNA3.1-D/EMCV IRES no uORF nLuc (SacI/XbaI)
GAGCTCTCTGGCTAACTAGAGAACCCACTGCTTACTGGCTTATCGAAATTAATACGACTCA
CTATAGGGAGACCCAAGCTGG**TTGGGGCGCGTGGTGGCGGCTGCAGCCGCCACCACGC**
GCCCCGGCTAGTTAAGCTTGGTACCGAGCTCGGATCCGCCCTCGAGCGGGATCAATTCCG

CCCCCCCCCTAACGTTACTGGCCGAAGCCGCTTGGAATAAGGCCGGTGTGCGTTTGTCTA
TATGTTATTTTCCACCATATTGCCGTCTTTTGGCAATGTGAGGGCCCGGAAACCTGGCCCT
GTCTTCTTGACGAGCATTCCTAGGGGTCTTTCCCCTCTCGCCAAAGGAATGCAAGGTCTGT
TGAATGTCGTGAAGGAAGCAGTTCCTCTGGAAGCTTCTTGAAGACAAACAACGTCTGTAGC
GACCCTTTGCAGGCAGCGGAACCCCCCACCTGGCGACAGGTGCCTCTGCGGCCAAAAGC
CACGTGTATAAGATACACCTGCAAAGGCGGCACAACCCCAGTGCCACGTTGTGAGTTGGA
TAGTTGTGGAAAGAGTCAAATGGCTCTCCTCAAGCGTATTCAACAAGGGGCTGAAGGATGC
CCAGAAGGTACCCCATTGTATGGGATCTGATCTGGGGCCTCGGTGCACATGCTTTACATGT
GTTTAGTCGAGGTTAAAAAACGTCTAGGCCCCCCGAACCACGGGGACGTGGTTTTCCTTTG
AAAAACACGatgataatATGGTCTTCACACTCGAAGATTTCGTTGGGGACTGGCGACAGACAG
CCGGCTACAACCTGGACCAAGTCCTTGAACAGGGAGGTGTGTCCAGTTTGTTTCAGAATCT
CGGGGTGTCCGTAACTCCGATCCAAAGGATTGTCCTGAGCGGTGAAAATGGGCTGAAGAT
CGACATCCATGTCATCATCCCGTATGAAGGTCTGAGCGGCGACCAAATGGGCCAGATCGA
AAAAATTTTTAAGGTGGTGTACCCTGTGGATGATCATCACTTTAAGGTGATCCTGCACTATG
GCACACTGGTAATCGACGGGGTTACGCCGAACATGATCGACTATTTCGGGCGGCCGTATG
AAGGCATCGCCGTGTTCGACGGCAAAAAGATCACTGTAACAGGGACCCTGTGGAACGGCA
ACAAAATTATCGACGAGCGCCTGATCAACCCCGACGGCTCCCTGCTGTTCCGAGTAACCAT
CAACGGAGTGACCGGCTGGCGGCTGTGCGAACGCATTCTGGCGGACTACAAAGACCATG
ACGGTGATTATAAAGATCATGACATCGATTACAAGGATGACGATGACAAGTAAAAGGGTCA
AGACAATTCTGCAGATATCCAGCACAGTGGCGGCCGCTCGAGTCTAGA

pcDNA3.1-D/EMCV IRES uORF nLuc (SacI/XbaI)_16 nt spacer
GAGCTCTCTGGCTAACTAGAGAACCCACTGCTTACTGGCTTATCGAAATTAATACGACTCA
CTATAGGGAGACCCAAGCTGG**TTGGGGCGCGTGGTGGCGGCTGCAGCCGCCACCACGC**
GCCCCGGCTAGTTAAGCTTGGTACCGAGCTCGGATCCGCCCTCGAGCGGGATCAATTCCG
CCCCCCCCCTAACGTTACTGGCCGAAGCCGCTTGGAATAAGGCCGGTGTGCGTTTGTCTA
TATGTTATTTTCCACCATATTGCCGTCTTTTGGCAATGTGAGGGCCCGGAAACCTGGCCCT
GTCTTCTTGACGAGCATTCCTAGGGGTCTTTCCCCTCTCGCCAAAGGAATGCAAGGTCTGT
TGAATGTCGTGAAGGAAGCAGTTCCTCTGGAAGCTTCTTGAAGACAAACAACGTCTGTAGC
GACCCTTTGCAGGCAGCGGAACCCCCCACCTGGCGACAGGTGCCTCTGCGGCCAAAAGC
CACGTGTATAAGATACACCTGCAAAGGCGGCACAACCCCAGTGCCACGTTGTGAGTTGGA
TAGTTGTGGAAAGAGTCAAATGGCTCTCCTCAAGCGTATTCAACAAGGGGCTGAAGGATGC
CCAGAAGGTACCCCATTGTATGGGATCTGATCTGGGGCCTCGGTGCACATGCTTTACATGT
GTTTAGTCGAGGTTAAAAAACGTCTAGGCCCCCCGAACCACGGGGACGTGGTTTTCCTTTG
AAAAACACGatgataatatggtcaccatggtcaccatggtctaaacaacaacaacaacaaATGGTCTTCACACTCG
AAGATTTCGTTGGGGACTGGCGACAGACAGCCGGCTACAACCTGGACCAAGTCCTTGAAC
AGGGAGGTGTGTCCAGTTTGTTTCAGAATCTCGGGGTGTCCGTAACTCCGATCCAAAGGAT
TGTCCTGAGCGGTGAAAATGGGCTGAAGATCGACATCCATGTCATCATCCCGTATGAAGGT
CTGAGCGGCGACCAAATGGGCCAGATCGAAAAAATTTTTAAGGTGGTGTACCCTGTGGAT
GATCATCACTTTAAGGTGATCCTGCACTATGGCACACTGGTAATCGACGGGGTTACGCCGA
ACATGATCGACTATTTCGGGCGGCCGTATGAAGGCATCGCCGTGTTCGACGGCAAAAAGA
TCACTGTAACAGGGACCCTGTGGAACGGCAACAAAATTATCGACGAGCGCCTGATCAACC
CCGACGGCTCCCTGCTGTTCCGAGTAACCATCAACGGAGTGACCGGCTGGCGGCTGTGC
GAACGCATTCTGGCGGACTACAAAGACCATGACGGTGATTATAAAGATCATGACATCGATT
ACAAGGATGACGATGACAAGTAAAAGGGTCAAGACAATTCTGCAGATATCCAGCACAGTGG
CGGCCGCTCGAGTCTAGA

pcDNA3.1-D/EMCV IRES uORF nLuc (SacI/XbaI)_115 nt spacer
GAGCTCTCTGGCTAACTAGAGAACCCACTGCTTACTGGCTTATCGAAATTAATACGACTCA
CTATAGGGAGACCCAAGCTGG**TTGGGGCGCGTGGTGGCGGCTGCAGCCGCCACCACGC**
GCCCCGGCTAGTTAAGCTTGGTACCGAGCTCGGATCCGCCCTCGAGCGGGATCAATTCCG

CCCCCCCCCTAACGTTACTGGCCGAAGCCGCTTGGAATAAGGCCGGTGTGCGTTTGTCTA
TATGTTATTTTCCACCATATTGCCGTCTTTTGGCAATGTGAGGGCCCGGAAACCTGGCCCT
GTCTTCTTGACGAGCATTCCTAGGGGTCTTTCCCCTCTCGCCAAAGGAATGCAAGGTCTGT
TGAATGTCGTGAAGGAAGCAGTTCCTCTGGAAGCTTCTTGAAGACAAACAACGTCTGTAGC
GACCCTTTGCAGGCAGCGGAACCCCCCACCTGGCGACAGGTGCCTCTGCGGCCAAAAGC
CACGTGTATAAGATACACCTGCAAAGGCGGCACAACCCCAGTGCCACGTTGTGAGTTGGA
TAGTTGTGGAAAGAGTCAAATGGCTCTCCTCAAGCGTATTCAACAAGGGGCTGAAGGATGC
CCAGAAGGTACCCCATTGTATGGGATCTGATCTGGGGCCTCGGTGCACATGCTTTACATGT
GTTTAGTCGAGGTTAAAAAACGTCTAGGCCCCCCGAACCACGGGGACGTGGTTTTCCTTTG
AAAAACACGatgataatatggtcaccatggtcaccatggtctaaacaacaacaacaacaacaacaacaacaacaacaacaacaacaa
caacaacaacaacaacaacaacaacaacaacaacaacaacaacaacaacaacaacaacaacaacaacaacaacaacaacaacaacaacaacaa
ATGGTCTTCACACTCGAAGATTTCGTTGGGGACTGGCGACAGACAGCCGGCTACAACCTG
GACCAAGTCCTTGAACAGGGAGGTGTGTCCAGTTTGTTTCAGAATCTCGGGGTGTCCGTAA
CTCCGATCCAAAGGATTGTCCTGAGCGGTGAAAATGGGCTGAAGATCGACATCCATGTCAT
CATCCCGTATGAAGGTCTGAGCGGCGACCAAATGGGCCAGATCGAAAAAATTTTTAAGGTG
GTGTACCCTGTGGATGATCATCACTTTAAGGTGATCCTGCACTATGGCACACTGGTAATCG
ACGGGGTTACGCCGAACATGATCGACTATTTCGGGCGGCCGTATGAAGGCATCGCCGTGT
TCGACGGCAAAAAGATCACTGTAACAGGGACCCTGTGGAACGGCAACAAAATTATCGACG
AGCGCCTGATCAACCCCGACGGCTCCCTGCTGTTCCGAGTAACCATCAACGGAGTGACCG
GCTGGCGGCTGTGCGAACGCATTCTGGCGGACTACAAAGACCATGACGGTGATTATAAAG
ATCATGACATCGATTACAAGGATGACGATGACAAGTAAAAGGGTCAAGACAATTCTGCAGA
TATCCAGCACAGTGGCGGCCGCTCGAGTCTAGA

pcDNA3.1-D/HCV no uORF nLuc (SacI/XbaI)
GAGCTCTCTGGCTAACTAGAGAACCCACTGCTTACTGGCTTATCGAAATTAATACGACTCA
CTATAGGGAGACCCAAGCTGG**TTGGGGCGCGTGGTGGCGGCTGCAGCCGCCACCACGC**
GCCCCGGCTAGTTAAGCTTGGTACCGAGCTCGGATCCCCTGTGAGGAACTACTGTCTTCA
CGCAGAAAGCGCCTAGCCATGGCGTTAGTATGAGTGTCGTACAGCCTCCAGGCCCCCCC
TCCCGGGAGAGCCATAGTGGTCTGCGGAACCGGTGAGTACACCGGAATTGCCGGGAAGA
CTGGGTCCTTTCTTGGATAAACCCACTCTATGCCCGGCCATTTGGGCGTGCCCCCGCAAG
ACTGCTAGCCGAGTAGCGTTGGGTTGCGAAAGGCCTTGTGGTACTGCCTGATAGGGCGCT
TGCGAGTGCCCCGGGAGGTCTCGTAGACCGTGCatcatgagcacgaatcctaaacctcaaagaaaaATG
GTCTTCACACTCGAAGATTTCGTTGGGGACTGGCGACAGACAGCCGGCTACAACCTGGAC
CAAGTCCTTGAACAGGGAGGTGTGTCCAGTTTGTTTCAGAATCTCGGGGTGTCCGTAACTC
CGATCCAAAGGATTGTCCTGAGCGGTGAAAATGGGCTGAAGATCGACATCCATGTCATCAT
CCCGTATGAAGGTCTGAGCGGCGACCAAATGGGCCAGATCGAAAAAATTTTTAAGGTGGT
GTACCCTGTGGATGATCATCACTTTAAGGTGATCCTGCACTATGGCACACTGGTAATCGAC
GGGGTTACGCCGAACATGATCGACTATTTCGGACGGCCGTATGAAGGCATCGCCGTGTTC
GACGGCAAAAAGATCACTGTAACAGGGACCCTGTGGAACGGCAACAAAATTATCGACGAG
CGCCTGATCAACCCCGACGGCTCCCTGCTGTTCCGAGTAACCATCAACGGAGTGACCGGC
TGGCGGCTGTGCGAACGCATTCTGGCGGACTACAAAGACCATGACGGTGATTATAAAGAT
CATGACATCGATTACAAGGATGACGATGACAAGTAAAAGGGTCAAGACAATTCTGCAGATA
TCCAGCACAGTGGCGGCCGCTCGAGTCTAGA

pcDNA3.1-D/HCV IRES uORF nLuc (SacI/XbaI)_16 nt spacer
GAGCTCTCTGGCTAACTAGAGAACCCACTGCTTACTGGCTTATCGAAATTAATACGACTCA
CTATAGGGAGACCCAAGCTGG**TTGGGGCGCGTGGTGGCGGCTGCAGCCGCCACCACGC**
GCCCCGGCTAGTTAAGCTTGGTACCGAGCTCGGATCCCCTGTGAGGAACTACTGTCTTCA
CGCAGAAAGCGCCTAGCCATGGCGTTAGTATGAGTGTCGTACAGCCTCCAGGCCCCCCC
TCCCGGGAGAGCCATAGTGGTCTGCGGAACCGGTGAGTACACCGGAATTGCCGGGAAGA
CTGGGTCCTTTCTTGGATAAACCCACTCTATGCCCGGCCATTTGGGCGTGCCCCCGCAAG

ACTGCTAGCCGAGTAGCGTTGGGTTGCGAAAGGCCTTGTGGTACTGCCTGATAGGGCGCT
TGCGAGTGCCCCGGGAGGTCTCGTAGACCGTGCatcatgagcacgaatcctaaacctcaaagaaaataaa
caacaacaacaacaaATGGTCTTCACACTCGAAGATTTCGTTGGGGACTGGCGACAGACAGCCG
GCTACAACCTGGACCAAGTCCTTGAACAGGGAGGTGTGTCCAGTTTGTTTCAGAATCTCGG
GGTGTCCGTAACTCCGATCCAAAGGATTGTCCTGAGCGGTGAAAATGGGCTGAAGATCGA
CATCCATGTCATCATCCCGTATGAAGGTCTGAGCGGCGACCAAATGGGCCAGATCGAAAA
AATTTTTAAGGTGGTGTACCCTGTGGATGATCATCACTTTAAGGTGATCCTGCACTATGGCA
CACTGGTAATCGACGGGGTTACGCCGAACATGATCGACTATTTCGGACGGCCGTATGAAG
GCATCGCCGTGTTCGACGGCAAAAAGATCACTGTAACAGGGACCCTGTGGAACGGCAACA
AAATTATCGACGAGCGCCTGATCAACCCCGACGGCTCCCTGCTGTTCCGAGTAACCATCAA
CGGAGTGACCGGCTGGCGGCTGTGCGAACGCATTCTGGCGGACTACAAAGACCATGACG
GTGATTATAAAGATCATGACATCGATTACAAGGATGACGATGACAAGTAAAAGGGTCAAGA
CAATTCTGCAGATATCCAGCACAGTGGCGGCCGCTCGAGTCTAGA

pcDNA3.1-D/HCV IRES uORF nLuc (SacI/XbaI)_115 nt spacer
GAGCTCTCTGGCTAACTAGAGAACCCACTGCTTACTGGCTTATCGAAATTAATACGACTCA
CTATAGGGAGACCCAAGCTGG**TTGGGGCGCGTGGTGGCGGCTGCAGCCGCCACCACGC**
GCCCCGGCTAGTTAAGCTTGGTACCGAGCTCGGATCCCCTGTGAGGAACTACTGTCTTCA
CGCAGAAAGCGCCTAGCCATGGCGTTAGTATGAGTGTCGTACAGCCTCCAGGCCCCCCC
TCCCGGGAGAGCCATAGTGGTCTGCGGAACCGGTGAGTACACCGGAATTGCCGGGAAGA
CTGGGTCCTTTCTTGGATAAACCCACTCTATGCCCGGCCATTTGGGCGTGCCCCCGCAAG
ACTGCTAGCCGAGTAGCGTTGGGTTGCGAAAGGCCTTGTGGTACTGCCTGATAGGGCGCT
TGCGAGTGCCCCGGGAGGTCTCGTAGACCGTGCatcatgagcacgaatcctaaacctcaaagaaaataaa
caacaacaacaacaacaacaacaacaacaacaacaacaacaacaacaacaacaacaacaacaacaacaacaacaacaac
aacaacaacaacaacaacaacaacaacaacaacaaATGGTCTTCACACTCGAAGATTTCGTTGGGGACT
GGCGACAGACAGCCGGCTACAACCTGGACCAAGTCCTTGAACAGGGAGGTGTGTCCAGTT
TGTTTCAGAATCTCGGGGTGTCCGTAACTCCGATCCAAAGGATTGTCCTGAGCGGTGAAAA
TGGGCTGAAGATCGACATCCATGTCATCATCCCGTATGAAGGTCTGAGCGGCGACCAAAT
GGGCCAGATCGAAAAAATTTTTAAGGTGGTGTACCCTGTGGATGATCATCACTTTAAGGTG
ATCCTGCACTATGGCACACTGGTAATCGACGGGGTTACGCCGAACATGATCGACTATTTCG
GACGGCCGTATGAAGGCATCGCCGTGTTCGACGGCAAAAAGATCACTGTAACAGGGACCC
TGTGGAACGGCAACAAAATTATCGACGAGCGCCTGATCAACCCCGACGGCTCCCTGCTGT
TCCGAGTAACCATCAACGGAGTGACCGGCTGGCGGCTGTGCGAACGCATTCTGGCGGAC
TACAAAGACCATGACGGTGATTATAAAGATCATGACATCGATTACAAGGATGACGATGACA
AGTAAAAGGGTCAAGACAATTCTGCAGATATCCAGCACAGTGGCGGCCGCTCGAGTCTAG
A

pcDNA3.1-D/CrPV IGR IRES no uORF nLuc (SacI/XbaI)
GAGCTCTCTGGCTAACTAGAGAACCCACTGCTTACTGGCTTATCGAAATTAATACGACTCA
CTATAGGGAGACCCAAGCTGG**TTGGGGCGCGTGGTGGCGGCTGCAGCCGCCACCACGC**
GCCCCGGCTAGTTAAGCTTGGTACCGAGCTCGGATCCAGTACCCTTCACCAAAGCAAAAT
GTGATCTTGCTTGTAAATACAATTTTGAGAGGTTAATAAATTACAAGTAGTGCTATTTTTGTA
TTTAGGTTAGCTATTTAGCTTTACGTTCCAGGATGCCTAGTGGCAGCCCCACAATATCCAG
GAAGCCCTCTCTGCGGTTTTTCAGATTAGGTAGTCGAAAAACCTAAGAAATTTACCTGCTAC
ATTTCAAGATACCATGGTCTTCACACTCGAAGATTTCGTTGGGGACTGGCGACAGACAGCC
GGCTACAACCTGGACCAAGTCCTTGAACAGGGAGGTGTGTCCAGTTTGTTTCAGAATCTCG
GGGTGTCCGTAACTCCGATCCAAAGGATTGTCCTGAGCGGTGAAAATGGGCTGAAGATCG
ACATCCATGTCATCATCCCGTATGAAGGTCTGAGCGGCGACCAAATGGGCCAGATCGAAA
AAATTTTTAAGGTGGTGTACCCTGTGGATGATCATCACTTTAAGGTGATCCTGCACTATGGC
ACACTGGTAATCGACGGGGTTACGCCGAACATGATCGACTATTTCGGACGGCCGTATGAA
GGCATCGCCGTGTTCGACGGCAAAAAGATCACTGTAACAGGGACCCTGTGGAACGGCAAC

AAAATTATCGACGAGCGCCTGATCAACCCCGACGGCTCCCTGCTGTTCCGAGTAACCATCA
ACGGAGTGACCGGCTGGCGGCTGTGCGAACGCATTCTGGCGGACTACAAAGACCATGAC
GGTGATTATAAAGATCATGACATCGATTACAAGGATGACGATGACAAGTAAAAGGGTCAAG
ACAATTCTGCAGATATCCAGCACAGTGGCGGCCGCTCGAGTCTAGA

pcDNA3.1-D/CrPV IGR IRES uORF nLuc (SacI/XbaI)_16 nt spacer
GAGCTCTCTGGCTAACTAGAGAACCCACTGCTTACTGGCTTATCGAAATTAATACGACTCA
CTATAGGGAGACCCAAGCTGG**TTGGGGCGCGTGGTGGCGGCTGCAGCCGCCACCACGC**
GCCCCGGCTAGTTAAGCTTGGTACCGAGCTCGGATCCAGTACCCTTCACCAAAGCAAAAT
GTGATCTTGCTTGTAAATACAATTTTGAGAGGTTAATAAATTACAAGTAGTGCTATTTTTGTA
TTTAGGTTAGCTATTTAGCTTTACGTTCCAGGATGCCTAGTGGCAGCCCCACAATATCCAG
GAAGCCCTCTCTGCGGTTTTTCAGATTAGGTAGTCGAAAAACCTAAGAAATTTACCTGCTAC
ATTTCAAGATACCatggtcaccatggtcaccatggtctaaacaacaacaacaacaaATGGTCTTCACACTCGAA
GATTTCGTTGGGGACTGGCGACAGACAGCCGGCTACAACCTGGACCAAGTCCTTGAACAG
GGAGGTGTGTCCAGTTTGTTTCAGAATCTCGGGGTGTCCGTAACTCCGATCCAAAGGATTG
TCCTGAGCGGTGAAAATGGGCTGAAGATCGACATCCATGTCATCATCCCGTATGAAGGTCT
GAGCGGCGACCAAATGGGCCAGATCGAAAAAATTTTTAAGGTGGTGTACCCTGTGGATGA
TCATCACTTTAAGGTGATCCTGCACTATGGCACACTGGTAATCGACGGGGTTACGCCGAAC
ATGATCGACTATTTCGGACGGCCGTATGAAGGCATCGCCGTGTTCGACGGCAAAAAGATC
ACTGTAACAGGGACCCTGTGGAACGGCAACAAAATTATCGACGAGCGCCTGATCAACCCC
GACGGCTCCCTGCTGTTCCGAGTAACCATCAACGGAGTGACCGGCTGGCGGCTGTGCGA
ACGCATTCTGGCGGACTACAAAGACCATGACGGTGATTATAAAGATCATGACATCGATTAC
AAGGATGACGATGACAAGTAAAAGGGTCAAGACAATTCTGCAGATATCCAGCACAGTGGC
GGCCGCTCGAGTCTAGA

pcDNA3.1-D/CrPV IGR IRES uORF nLuc (SacI/XbaI)_115 nt spacer
GAGCTCTCTGGCTAACTAGAGAACCCACTGCTTACTGGCTTATCGAAATTAATACGACTCA
CTATAGGGAGACCCAAGCTGG**TTGGGGCGCGTGGTGGCGGCTGCAGCCGCCACCACGC**
GCCCCGGCTAGTTAAGCTTGGTACCGAGCTCGGATCCAGTACCCTTCACCAAAGCAAAAT
GTGATCTTGCTTGTAAATACAATTTTGAGAGGTTAATAAATTACAAGTAGTGCTATTTTTGTA
TTTAGGTTAGCTATTTAGCTTTACGTTCCAGGATGCCTAGTGGCAGCCCCACAATATCCAG
GAAGCCCTCTCTGCGGTTTTTCAGATTAGGTAGTCGAAAAACCTAAGAAATTTACCTGCTAC
ATTTCAAGATACCatggtcaccatggtcaccatggtctaaacaacaacaacaacaacaacaacaacaacaacaacaaca
acaacaacaacaacaacaacaacaacaacaacaacaacaacaacaacaacaacaacaacaacaacaacaacaacaacaaATGG
TCTTCACACTCGAAGATTTCGTTGGGGACTGGCGACAGACAGCCGGCTACAACCTGGACC
AAGTCCTTGAACAGGGAGGTGTGTCCAGTTTGTTTCAGAATCTCGGGGTGTCCGTAACTCC
GATCCAAAGGATTGTCCTGAGCGGTGAAAATGGGCTGAAGATCGACATCCATGTCATCATC
CCGTATGAAGGTCTGAGCGGCGACCAAATGGGCCAGATCGAAAAAATTTTTAAGGTGGTG
TACCCTGTGGATGATCATCACTTTAAGGTGATCCTGCACTATGGCACACTGGTAATCGACG
GGGTTACGCCGAACATGATCGACTATTTCGGACGGCCGTATGAAGGCATCGCCGTGTTCG
ACGGCAAAAAGATCACTGTAACAGGGACCCTGTGGAACGGCAACAAAATTATCGACGAGC
GCCTGATCAACCCCGACGGCTCCCTGCTGTTCCGAGTAACCATCAACGGAGTGACCGGCT
GGCGGCTGTGCGAACGCATTCTGGCGGACTACAAAGACCATGACGGTGATTATAAAGATC
ATGACATCGATTACAAGGATGACGATGACAAGTAAAAGGGTCAAGACAATTCTGCAGATAT
CCAGCACAGTGGCGGCCGCTCGAGTCTAGA

pTet-Off AIO/no uORF nLuc (MluI/XbaI)
ACGCGTTgctcgagcactttggccgcgaatcgatatgtcgagtttactccctatcagtgatagagaacgtatgtcgagtttactccct
atcagtgatagagaacgatgtcgagtttactccctatcagtgatagagaacgtatgtcgagtttactccctatcagtgatagagaacgtat
gtcgagtttactccctatcagtgatagagaacgtatgtcgagtttatccctatcagtgatagagaacgtatgtcgagtttactccctatcagt
gatagagaacgtatgtcgaggtaggcgtgtacggtgggaggcctatataagcaGAGCTCTCTGGCTAACTAGAGAA

CCCACTGCTTACTGGCTTATCGAAATTAATACGACTCACTATAGGcaacaacaacaacaacaacaac
aacaacaacaacaacaacaacaacaacaacaacaacaacaacaacaacaaATGGTCTTCACACTCGAAGATTT
CGTTGGGGACTGGCGACAGACAGCCGGCTACAACCTGGACCAAGTCCTTGAACAGGGAG
GTGTGTCCAGTTTGTTTCAGAATCTCGGGGTGTCCGTAACTCCGATCCAAAGGATTGTCCT
GAGCGGTGAAAATGGGCTGAAGATCGACATCCATGTCATCATCCCGTATGAAGGTCTGAG
CGGCGACCAAATGGCCCAGATCGAAAAAATTTTTAAGGTGGTGTACCCTGTGGATGATCAT
CACTTTAAGGTGATCCTGCACTATGGCACACTGGTAATCGACGGGGTTACGCCGAACATGA
TCGACTATTTCGGACGGCCGTATGAAGGCATCGCCGTGTTCGACGGCAAAAAGATCACTG
TAACAGGGACCCTGTGGAACGGCAACAAAATTATCGACGTAAGTATCAAGGTTACAAGACA
GGTTTAAGGAGACCAATAGAAACTGGGCTTGTCGAGACAGAGAAGACTCTTGCGTTTCTGA
TAGGCACCTATTGGTCTTACTGACATCCACTTTGCCTTTCTCTCCACAGGAGCGCCTGATC
AACCCCGACGGCTCCCTGCTGTTCCGAGTAACCATCAACGGAGTGACCGGCTGGCGGCT
GTGCGAACGCATTCTGGCGGACTACAAAGACCATGACGGTGATTATAAAGATCATGACATC
GATTACAAGGATGACGATGACAAGTAAGGCCGCGACTCTAGA

pTet-Off AIO/no uORF nLuc + 3' UTR intron (MluI/PflMI)

ACGCGTTgctcgagcactttggccgcgaatcgatatgtcgagtttactccctatcagtgatagagaacgtatgtcgagtttactccct
atcagtgatagagaacgatgtcgagtttactccctatcagtgatagagaacgtatgtcgagtttactccctatcagtgatagagaacgtat
gtcgagtttactccctatcagtgatagagaacgtatgtcgagtttatccctatcagtgatagagaacgtatgtcgagtttactccctatcagt
gatagagaacgtatgtcgaggtaggcgtgtacggtgggaggcctatataagcaGAGCTCTCTGGCTAACTAGAGAA
CCCACTGCTTACTGGCTTATCGAAATTAATACGACTCACTATAGGcaacaacaacaacaacaacaac
aacaacaacaacaacaacaacaacaacaacaacaacaacaacaacaacaaATGGTCTTCACACTCGAAGATTT
CGTTGGGGACTGGCGACAGACAGCCGGCTACAACCTGGACCAAGTCCTTGAACAGGGAG
GTGTGTCCAGTTTGTTTCAGAATCTCGGGGTGTCCGTAACTCCGATCCAAAGGATTGTCCT
GAGCGGTGAAAATGGGCTGAAGATCGACATCCATGTCATCATCCCGTATGAAGGTCTGAG
CGGCGACCAAATGGCCCAGATCGAAAAAATTTTTAAGGTGGTGTACCCTGTGGATGATCAT
CACTTTAAGGTGATCCTGCACTATGGCACACTGGTAATCGACGGGGTTACGCCGAACATGA
TCGACTATTTCGGACGGCCGTATGAAGGCATCGCCGTGTTCGACGGCAAAAAGATCACTG
TAACAGGGACCCTGTGGAACGGCAACAAAATTATCGACGTAAGTATCAAGGTTACAAGACA
GGTTTAAGGAGACCAATAGAAACTGGGCTTGTCGAGACAGAGAAGACTCTTGCGTTTCTGA
TAGGCACCTATTGGTCTTACTGACATCCACTTTGCCTTTCTCTCCACAGGAGCGCCTGATC
AACCCCGACGGCTCCCTGCTGTTCCGAGTAACCATCAACGGAGTGACCGGCTGGCGGCT
GTGCGAACGCATTCTGGCGGACTACAAAGACCATGACGGTGATTATAAAGATCATGACATC
GATTACAAGGATGACGATGACAAGTAAGGCCGCGACTCTAGAGGGCCCGTTTAAACCCGC
TGATCAGCCTCGAGCAGAAATCGGTACTGGCTTTCCATTCGACGTAAGTATCAAGGTTACA
AGACAGGTTTAAGGAGACCAATAGAAACTGGGCTTGTCGAGACAGAGAAGACTCTTGCGTT
TCTGATAGGCACCTATTGGTCTTACTGACATCCACTTTGCCTTTCTCTCCACAGCCCCATTA
TGTGG

pTet-Off AIO/uORF nLuc (MluI/XbaI)

ACGCGTTgctcgagcactttggccgcgaatcgatatgtcgagtttactccctatcagtgatagagaacgtatgtcgagtttactccct
atcagtgatagagaacgatgtcgagtttactccctatcagtgatagagaacgtatgtcgagtttactccctatcagtgatagagaacgtat
gtcgagtttactccctatcagtgatagagaacgtatgtcgagtttatccctatcagtgatagagaacgtatgtcgagtttactccctatcagt
gatagagaacgtatgtcgaggtaggcgtgtacggtgggaggcctatataagcaGAGCTCTCTGGCTAACTAGAGAA
CCCACTGCTTACTGGCTTATCGAAATTAATACGACTCACTATAGGcaacaacaacaacaacaacaac
aacaacaacaacaacaacaacaacaacaacaacaacaacaacaacaacaccatggtcaccatggtcaccatggtctaaa
caacaacaacaacaaATGGTCTTCACACTCGAAGATTTCGTTGGGGACTGGCGACAGACAGCCG
GCTACAACCTGGACCAAGTCCTTGAACAGGGAGGTGTGTCCAGTTTGTTTCAGAATCTCGG
GGTGTCCGTAACTCCGATCCAAAGGATTGTCCTGAGCGGTGAAAATGGGCTGAAGATCGA
CATCCATGTCATCATCCCGTATGAAGGTCTGAGCGGCGACCAAATGGCCCAGATCGAAAA
AATTTTTAAGGTGGTGTACCCTGTGGATGATCATCACTTTAAGGTGATCCTGCACTATGGCA

CACTGGTAATCGACGGGGTTACGCCGAACATGATCGACTATTTCGGACGGCCGTATGAAG
GCATCGCCGTGTTCGACGGCAAAAAGATCACTGTAACAGGGACCCTGTGGAACGGCAACA
AAATTATCGACGTAAGTATCAAGGTTACAAGACAGGTTTAAGGAGACCAATAGAAACTGGG
CTTGTCGAGACAGAGAAGACTCTTGCGTTTCTGATAGGCACCTATTGGTCTTACTGACATC
CACTTTGCCTTTCTCTCCACAGGAGCGCCTGATCAACCCCGACGGCTCCCTGCTGTTCCG
AGTAACCATCAACGGAGTGACCGGCTGGCGGCTGTGCGAACGCATTCTGGCGGACTACAA
AGACCATGACGGTGATTATAAAGATCATGACATCGATTACAAGGATGACGATGACAAGTAA
GGCCGCGACTCTAGA

pTet-Off AIO/large uORF nLuc (MluI/XbaI)
ACGCGTTgctcgagcactttggccgcgaatcgatatgtcgagtttactccctatcagtgatagagaacgtatgtcgagtttactccct
atcagtgatagagaacgatgtcgagtttactccctatcagtgatagagaacgtatgtcgagtttactccctatcagtgatagagaacgtat
gtcgagtttactccctatcagtgatagagaacgtatgtcgagtttatccctatcagtgatagagaacgtatgtcgagtttactccctatcagt
gatagagaacgtatgtcgaggtaggcgtgtacggtgggaggcctatataagcaGAGCTCTCTGGCTAACTAGAGAA
CCCACTGCTTACTGGCTTATCGAAATTAATACGACTCACTATAGGaacaacaacaacaacaacaac
aacaacaacaacaacaacaacaacaacaacaacaacaacaacaacaacaacaccatggtcaccatggtcaccatggtcGCA
GAAATCGGTACTGGCTTTCCATTCGACCCCCATTATGTGGAAGTCCTGGGCGAGCGCATG
CACTACGTCGATGTTGGTCCGCGCGATGGCACCCCTGTGCTGTTCCTGCACGGTAACCCG
ACCTCCTCCTACGTGTGGCGCAACATCATCCCGCATGTTGCACCGACCCATCGCTGCATT
GCTCCAGACCTGATCGGTATGGGCAAATCCGACAAACCAGACCTGGGTTATTTCTTCGACG
ACCACGTCCGCTTCATGGATGCCTTCATCGAAGCCCTGGGTCTGGAAGAGGTCGTCCTGG
TCATTCACGACTGGGGCTCCGCTCTGGGTTTCCACTGGGCCAAGCGCAATCCAGAGCGCG
TCAAAGGTATTGCATTTATGGAGTTCATCCGCCCTATCCCGACCTGGGACGAATGGCCAGA
ATTTGCCCGCGAGACCTTCCAGGCCTTCCGCACCACCGACGTCGGCCGCAAGCTGATCAT
CGATCAGAACGTTTTTATCGAGGGTACGCTGCCGATGGGTGTCGTCCGCCCGCTGACTGA
AGTCGAGATGGACCATTACCGCGAGCCGTTCCTGAATCCTGTTGACCGCGAGCCACTGTG
GCGCTTCCCAAACGAGCTGCCAATCGCCGGTGAGCCAGCGAACATCGTCGCGCTGGTCG
AAGAATACATGGACTGGCTGCACCAGTCCCCTGTCCCGAAGCTGCTGTTCTGGGGCACCC
CAGGCGTTCTGATCCCACCGGCCGAAGCCGCTCGCCTGGCCAAAAGCCTGCCTAACTGCA
AGGCTGTGGACATCGGCCCGGGTCTGAATCTGCTGCAAGAAGACAACCCGGACCTGATCG
GCAGCGAGATCGCGCGCTGGCTGTCGACGCTCGAGATTTCCGGCCACCGGTCGCAACCT
TGGGTACCGCGGGCCCGGGATCCACCGGTCGCAACCTTGGTGAGCAAGGGCGAGGAGCT
GTTCACCGGGGTGGTGCCCATCCTGGTCGAGCTGGACGGCGACGTAAACGGCCACAAGT
TCAGCGTGTCCGGCGAGGGCGAGGGCGATGCCACCTACGGCAAGCTGACCCTGAAGTTC
ATCTGCACCACCGGCAAGCTGCCCGTGCCCTGGCCCACCCTCGTGACCACCCTGACCTAC
GGCGTGCAGTGCTTCAGCCGCTACCCCGACCACATGAAGCAGCACGACTTCTTCAAGTCC
GCCATGCCCGAAGGCTACGTCCAGGAGCGCACCATCTTCTTCAAGGACGACGGCAACTAC
AAGACCCGCGCCGAGGTGAAGTTCGAGGGCGACACCCTGGTGAACCGCATCGAGCTGAA
GGGCATCGACTTCAAGGAGGACGGCAACATCCTGGGGCACAAGCTGGAGTACAACTACAA
CAGCCACAACGTCTATATCATGGCCGACAAGCAGAAGAACGGCATCAAGGTGAACTTCAA
GATCCGCCACAACATCGAGGACGGCAGCGTGCAGCTCGCCGACCACTACCAGCAGAACA
CCCCCATCGGCGACGGCCCCGTGCTGCTGCCCGACAACCACTACCTGAGCACCCAGTCC
GCCCTGAGCAAAGACCCCAACGAGAAGCGCGATCACATGGTCCTGCTGGAGTTCGTGACC
GCCGCCGGGATCACTCTCGGCATGGACGAGCTGTACAAGtaaacaacaacaacaacaaATGGTC
TTCACACTCGAAGATTTCGTTGGGGACTGGCGACAGACAGCCGGCTACAACCTGGACCAA
GTCCTTGAACAGGGAGGTGTGTCCAGTTTGTTTCAGAATCTCGGGGTGTCCGTAACTCCGA
TCCAAAGGATTGTCCTGAGCGGTGAAAATGGGCTGAAGATCGACATCCATGTCATCATCCC
GTATGAAGGTCTGAGCGGCGACCAAATGGGCCAGATCGAAAAAATTTTTAAGGTGGTGTAC
CCTGTGGATGATCATCACTTTAAGGTGATCCTGCACTATGGCACACTGGTAATCGACGGGG
TTACGCCGAACATGATCGACTATTTCGGACGGCCGTATGAAGGCATCGCCGTGTTCGACG
GCAAAAAGATCACTGTAACAGGGACCCTGTGGAACGGCAACAAAATTATCGACGTAAGTAT

CAAGGTTACAAGACAGGTTTAAGGAGACCAATAGAAACTGGGCTTGTCGAGACAGAGAAG
ACTCTTGCGTTTCTGATAGGCACCTATTGGTCTTACTGACATCCACTTTGCCTTTCTCTCCA
CAGGAGCGCCTGATCAACCCCGACGGCTCCCTGCTGTTCCGAGTAACCATCAACGGAGTG
ACCGGCTGGCGGCTGTGCGAACGCATTCTGGCGGACTACAAAGACCATGACGGTGATTAT
AAAGATCATGACATCGATTACAAGGATGACGATGACAAGTAAGGCCGCGACTCTAGA

pTet-Off AIO/HP + no uORF nLuc (MluI/XbaI)
ACGCGTTgctcgagcactttggccgcgaatcgatatgtcgagtttactccctatcagtgatagagaacgtatgtcgagtttactccct
atcagtgatagagaacgatgtcgagtttactccctatcagtgatagagaacgtatgtcgagtttactccctatcagtgatagagaacgtat
gtcgagtttactccctatcagtgatagagaacgtatgtcgagtttatccctatcagtgatagagaacgtatgtcgagtttactccctatcagt
gatagagaacgtatgtcgaggtaggcgtgtacggtgggaggcctatataagcaGAGCTCacatttgcttctgacacaactgtgttc
actagcaacctcaaacagacacc**TTGGGGCGCGTGGTGGCGGCTGCAGCCGCCACCACGCGCCCC**
GGTCTGGCTAACTAGAGAACCCACTGCTTACTGGCTTATCGAAATTAATACGACTCACTATA
GGcaacaacaacaacaacaacaacaacaacaacaacaacaacaacaacaacaacaacaacaacaacaacaaATG
GTCTTCACACTCGAAGATTTCGTTGGGGACTGGCGACAGACAGCCGGCTACAACCTGGAC
CAAGTCCTTGAACAGGGAGGTGTGTCCAGTTTGTTTCAGAATCTCGGGGTGTCCGTAACTC
CGATCCAAAGGATTGTCCTGAGCGGTGAAAATGGGCTGAAGATCGACATCCATGTCATCAT
CCCGTATGAAGGTCTGAGCGGCGACCAAATGGGCCAGATCGAAAAAATTTTTAAGGTGGT
GTACCCTGTGGATGATCATCACTTTAAGGTGATCCTGCACTATGGCACACTGGTAATCGAC
GGGGTTACGCCGAACATGATCGACTATTTCGGACGGCCGTATGAAGGCATCGCCGTGTTC
GACGGCAAAAAGATCACTGTAACAGGGACCCTGTGGAACGGCAACAAAATTATCGACGTA
AGTATCAAGGTTACAAGACAGGTTTAAGGAGACCAATAGAAACTGGGCTTGTCGAGACAGA
GAAGACTCTTGCGTTTCTGATAGGCACCTATTGGTCTTACTGACATCCACTTTGCCTTTCTC
TCCACAGGAGCGCCTGATCAACCCCGACGGCTCCCTGCTGTTCCGAGTAACCATCAACGG
AGTGACCGGCTGGCGGCTGTGCGAACGCATTCTGGCGGACTACAAAGACCATGACGGTG
ATTATAAAGATCATGACATCGATTACAAGGATGACGATGACAAGTAAGGCCGCGACTCTAG
A

pTet-Off AIO/HP + no uORF nLuc + 3' UTR intron (MluI/PflMI)
ACGCGTTgctcgagcactttggccgcgaatcgatatgtcgagtttactccctatcagtgatagagaacgtatgtcgagtttactccct
atcagtgatagagaacgatgtcgagtttactccctatcagtgatagagaacgtatgtcgagtttactccctatcagtgatagagaacgtat
gtcgagtttactccctatcagtgatagagaacgtatgtcgagtttatccctatcagtgatagagaacgtatgtcgagtttactccctatcagt
gatagagaacgtatgtcgaggtaggcgtgtacggtgggaggcctatataagcaGAGCTCacatttgcttctgacacaactgtgttc
actagcaacctcaaacagacacc**TTGGGGCGCGTGGTGGCGGCTGCAGCCGCCACCACGCGCCCC**
GGTCTGGCTAACTAGAGAACCCACTGCTTACTGGCTTATCGAAATTAATACGACTCACTATA
GGcaacaacaacaacaacaacaacaacaacaacaacaacaataacaacaacaacaacaacaacaacaacaacaaATG
GTCTTCACACTCGAAGATTTCGTTGGGGACTGGCGACAGACAGCCGGCTACAACCTGGAC
CAAGTCCTTGAACAGGGAGGTGTGTCCAGTTTGTTTCAGAATCTCGGGGTGTCCGTAACTC
CGATCCAAAGGATTGTCCTGAGCGGTGAAAATGGGCTGAAGATCGACATCCATGTCATCAT
CCCGTATGAAGGTCTGAGCGGCGACCAAATGGGCCAGATCGAAAAAATTTTTAAGGTGGT
GTACCCTGTGGATGATCATCACTTTAAGGTGATCCTGCACTATGGCACACTGGTAATCGAC
GGGGTTACGCCGAACATGATCGACTATTTCGGACGGCCGTATGAAGGCATCGCCGTGTTC
GACGGCAAAAAGATCACTGTAACAGGGACCCTGTGGAACGGCAACAAAATTATCGACGTA
AGTATCAAGGTTACAAGACAGGTTTAAGGAGACCAATAGAAACTGGGCTTGTCGAGACAGA
GAAGACTCTTGCGTTTCTGATAGGCACCTATTGGTCTTACTGACATCCACTTTGCCTTTCTC
TCCACAGGAGCGCCTGATCAACCCCGACGGCTCCCTGCTGTTCCGAGTAACCATCAACGG
AGTGACCGGCTGGCGGCTGTGCGAACGCATTCTGGCGGACTACAAAGACCATGACGGTG
ATTATAAAGATCATGACATCGATTACAAGGATGACGATGACAAGTAAGGCCGCGACTCTAG
AGGGCCCGTTTAAACCCGCTGATCAGCCTCGAGCAGAAATCGGTACTGGCTTTCCATTCG
ACGTAAGTATCAAGGTTACAAGACAGGTTTAAGGAGACCAATAGAAACTGGGCTTGTCGAG

ACAGAGAAGACTCTTGCGTTTCTGATAGGCACCTATTGGTCTTACTGACATCCACTTTGCCT
TTCTCTCCACAGCCCCATTATGTGG

pTet-Off AIO/HP + uORF nLuc (MluI/XbaI)

ACGCGTTgctcgagcactttggccgcgaatcgatatgtcgagtttactccctatcagtgatagagaacgtatgtcgagtttactccct
atcagtgatagagaacgatgtcgagtttactccctatcagtgatagagaacgtatgtcgagtttactccctatcagtgatagagaacgtat
gtcgagtttactccctatcagtgatagagaacgtatgtcgagtttatccctatcagtgatagagaacgtatgtcgagtttactccctatcagt
gatagagaacgtatgtcgaggtaggcgtgtacggtgggaggcctatataagcaGAGCTCacatttgcttctgacacaactgtgttc
actagcaacctcaaacagacacc**TTGGGGCGCGTGGTGGCGGCTGCAGCCGCCACCACGCGCCCC**
GGTCTGGCTAACTAGAGAACCCACTGCTTACTGGCTTATCGAAATTAATACGACTCACTATA
GGcaacaacaacaacaacaacaacaacaacaacaacaacaacaacaacaacaacaacaacaacaacaacaacaacaacacc
atggtcaccatggtcaccatggtctaaacaacaacaacaacaaATGGTCTTCACACTCGAAGATTTCGTTGGG
GACTGGCGACAGACAGCCGGCTACAACCTGGACCAAGTCCTTGAACAGGGAGGTGTGTC
CAGTTTGTTTCAGAATCTCGGGGTGTCCGTAACTCCGATCCAAAGGATTGTCCTGAGCGGT
GAAAATGGGCTGAAGATCGACATCCATGTCATCATCCCGTATGAAGGTCTGAGCGGCGAC
CAAATGGGCCAGATCGAAAAAATTTTTAAGGTGGTGTACCCTGTGGATGATCATCACTTTAA
GGTGATCCTGCACTATGGCACACTGGTAATCGACGGGGTTACGCCGAACATGATCGACTA
TTTCGGACGGCCGTATGAAGGCATCGCCGTGTTCGACGGCAAAAAGATCACTGTAACAGG
GACCCTGTGGAACGGCAACAAAATTATCGACGTAAGTATCAAGGTTACAAGACAGGTTTAA
GGAGACCAATAGAAACTGGGCTTGTCGAGACAGAGAAGACTCTTGCGTTTCTGATAGGCA
CCTATTGGTCTTACTGACATCCACTTTGCCTTTCTCTCCACAGGAGCGCCTGATCAACCCC
GACGGCTCCCTGCTGTTCCGAGTAACCATCAACGGAGTGACCGGCTGGCGGCTGTGCGA
ACGCATTCTGGCGGACTACAAAGACCATGACGGTGATTATAAAGATCATGACATCGATTAC
AAGGATGACGATGACAAGTAAGGCCGCGACTCTAGA

pTet-Off AIO/HP + large uORF nLuc (MluI/XbaI)

ACGCGTTgctcgagcactttggccgcgaatcgatatgtcgagtttactccctatcagtgatagagaacgtatgtcgagtttactccct
atcagtgatagagaacgatgtcgagtttactccctatcagtgatagagaacgtatgtcgagtttactccctatcagtgatagagaacgtat
gtcgagtttactccctatcagtgatagagaacgtatgtcgagtttatccctatcagtgatagagaacgtatgtcgagtttactccctatcagt
gatagagaacgtatgtcgaggtaggcgtgtacggtgggaggcctatataagcaGAGCTCacatttgcttctgacacaactgtgttc
actagcaacctcaaacagacacc**TTGGGGCGCGTGGTGGCGGCTGCAGCCGCCACCACGCGCCCC**
GGTCTGGCTAACTAGAGAACCCACTGCTTACTGGCTTATCGAAATTAATACGACTCACTATA
GGaacaacaacaacaacaacaacaacaacaacaacaacaacaacaacaacaacaacaacaacaacaacaacaacaacacca
tggtcaccatggtcaccatggtcGCAGAAATCGGTACTGGCTTTCCATTCGACCCCCATTATGTGGAA
GTCCTGGGCGAGCGCATGCACTACGTCGATGTTGGTCCGCGCGATGGCACCCCTGTGCT
GTTCCTGCACGGTAACCCGACCTCCTCCTACGTGTGGCGCAACATCATCCCGCATGTTGC
ACCGACCCATCGCTGCATTGCTCCAGACCTGATCGGTATGGGCAAATCCGACAAACCAGA
CCTGGGTTATTTCTTCGACGACCACGTCCGCTTCATGGATGCCTTCATCGAAGCCCTGGGT
CTGGAAGAGGTCGTCCTGGTCATTCACGACTGGGGCTCCGCTCTGGGTTTCCACTGGGCC
AAGCGCAATCCAGAGCGCGTCAAAGGTATTGCATTTATGGAGTTCATCCGCCCTATCCCGA
CCTGGGACGAATGGCCAGAATTTGCCCGCGAGACCTTCCAGGCCTTCCGCACCACCGAC
GTCGGCCGCAAGCTGATCATCGATCAGAACGTTTTTATCGAGGGTACGCTGCCGATGGGT
GTCGTCCGCCCGCTGACTGAAGTCGAGATGGACCATTACCGCGAGCCGTTCCTGAATCCT
GTTGACCGCGAGCCACTGTGGCGCTTCCCAAACGAGCTGCCAATCGCCGGTGAGCCAGC
GAACATCGTCGCGCTGGTCGAAGAATACATGGACTGGCTGCACCAGTCCCCTGTCCCGAA
GCTGCTGTTCTGGGGCACCCCAGGCGTTCTGATCCCACCGGCCGAAGCCGCTCGCCTGG
CCAAAAGCCTGCCTAACTGCAAGGCTGTGGACATCGGCCCGGGTCTGAATCTGCTGCAAG
AAGACAACCCGGACCTGATCGGCAGCGAGATCGCGCGCTGGCTGTCGACGCTCGAGATT
TCCGGCCACCGGTCGCAACCTTGGGTACCGCGGGCCCGGGATCCACCGGTCGCAACCTT
GGTGAGCAAGGGCGAGGAGCTGTTCACCGGGGTGGTGCCCATCCTGGTCGAGCTGGACG
GCGACGTAAACGGCCACAAGTTCAGCGTGTCCGGCGAGGGCGAGGGCGATGCCACCTAC

GGCAAGCTGACCCTGAAGTTCATCTGCACCACCGGCAAGCTGCCCGTGCCCTGGCCCAC
CCTCGTGACCACCCTGACCTACGGCGTGCAGTGCTTCAGCCGCTACCCCGACCACATGAA
GCAGCACGACTTCTTCAAGTCCGCCATGCCCGAAGGCTACGTCCAGGAGCGCACCATCTT
CTTCAAGGACGACGGCAACTACAAGACCCGCGCCGAGGTGAAGTTCGAGGGCGACACCC
TGGTGAACCGCATCGAGCTGAAGGGCATCGACTTCAAGGAGGACGGCAACATCCTGGGG
CACAAGCTGGAGTACAACTACAACAGCCACAACGTCTATATCATGGCCGACAAGCAGAAGA
ACGGCATCAAGGTGAACTTCAAGATCCGCCACAACATCGAGGACGGCAGCGTGCAGCTCG
CCGACCACTACCAGCAGAACACCCCCATCGGCGACGGCCCCGTGCTGCTGCCCGACAAC
CACTACCTGAGCACCCAGTCCGCCCTGAGCAAAGACCCCAACGAGAAGCGCGATCACATG
GTCCTGCTGGAGTTCGTGACCGCCGCCGGGATCACTCTCGGCATGGACGAGCTGTACAA
Gtaaacaacaacaacaacaacaaa ATGGTCTTCACACTCGAAGATTTCGTTGGGGACTGGCGACAGACA
GCCGGCTACAACCTGGACCAAGTCCTTGAACAGGGAGGTGTGTCCAGTTTGTTTCAGAAT
CTCGGGGTGTCCGTAACTCCGATCCAAAGGATTGTCCTGAGCGGTGAAAATGGGCTGAAG
ATCGACATCCATGTCATCATCCCGTATGAAGGTCTGAGCGGCGACCAAATGGGCCAGATC
GAAAAAATTTTTAAGGTGGTGTACCCTGTGGATGATCATCACTTTAAGGTGATCCTGCACTA
TGGCACACTGGTAATCGACGGGGTTACGCCGAACATGATCGACTATTTCGGACGGCCGTA
TGAAGGCATCGCCGTGTTCGACGGCAAAAAGATCACTGTAACAGGGACCCTGTGGAACGG
CAACAAAATTATCGACGTAAGTATCAAGGTTACAAGACAGGTTTAAGGAGACCAATAGAAA
CTGGGCTTGTCGAGACAGAGAAGACTCTTGCGTTTCTGATAGGCACCTATTGGTCTTACTG
ACATCCACTTTGCCTTTCTCTCCACAGGAGCGCCTGATCAACCCCGACGGCTCCCTGCTGT
TCCGAGTAACCATCAACGGAGTGACCGGCTGGCGGCTGTGCGAACGCATTCTGGCGGAC
TACAAAGACCATGACGGTGATTATAAAGATCATGACATCGATTACAAGGATGACGATGACA
AGTAAGGCCGCGACTCTAGA

pTet-Off AIO/no uORF nLuc + 3' UTR intron (MluI/PflMI)_stop mutated

ACGCGTTgctcgagcactttggccgcgaatcgatatgtcgagtttactccctatcagtgatagagaacgtatgtcgagtttactccct
atcagtgatagagaacgatgtcgagtttactccctatcagtgatagagaacgtatgtcgagtttactccctatcagtgatagagaacgtat
gtcgagtttactccctatcagtgatagagaacgtatgtcgagtttatccctatcagtgatagagaacgtatgtcgagtttactccctatcagt
gatagagaacgtatgtcgaggtaggcgtgtacggtgggaggcctatataagcaGAGCTCTCTGGCTAACTAGAGAA
CCCACTGCTTACTGGCTTATCGAAATTAATACGACTCACTATAGGcaacaacaacaacaacaacaac
aacaacaacaacaacaacaacaacaacaacaacaacaacaacaacaaATGGTCTTCACACTCGAAGATTT
CGTTGGGGACTGGCGACAGACAGCCGGCTACAACCTGGACCAAGTCCTTGAACAGGGAG
GTGTGTCCAGTTTGTTTCAGAATCTCGGGGTGTCCGTAACTCCGATCCAAAGGATTGTCCT
GAGCGGTGAAAATGGGCTGAAGATCGACATCCATGTCATCATCCCGTATGAAGGTCTGAG
CGGCGACCAAATGGGCCAGATCGAAAAAATTTTTAAGGTGGTGTACCCTGTGGATGATCAT
CACTTTAAGGTGATCCTGCACTATGGCACACTGGTAATCGACGGGGTTACGCCGAACATGA
TCGACTATTTCGGACGGCCGTATGAAGGCATCGCCGTGTTCGACGGCAAAAAGATCACTG
TAACAGGGACCCTGTGGAACGGCAACAAAATTATCGACGTAAGTATCAAGGTTACAAGACA
GGTTTAAGGAGACCAATAGAAACTGGGCTTGTCGAGACAGAGAAGACTCTTGCGTTTCTGA
TAGGCACCTATTGGTCTTACTGACATCCACTTTGCCTTTCTCTCCACAGGAGCGCCTGATC
AACCCCGACGGCTCCCTGCTGTTCCGAGTAACCATCAACGGAGTGACCGGCTGGCGGCT
GTGCGAACGCATTCTGGCGGACTACAAAGACCATGACGGTGATTATAAAGATCATGACATC
GATTACAAGGATGACGATGACAAGTACGGCCGCGACTCTACAGGGCCCGTTTTTACCCGC
TTTTCAGCCTCGAGCAGAAATCGGTACTGGCTTTCCATTCGACGTAAGTATCAAGGTTACA
AGACAGGTTTAAGGAGACCAATAGAAACTGGGCTTGTCGAGACAGAGAAGACTCTTGCGTT
TCTGATAGGCACCTATTGGTCTTACTGACATCCACTTTGCCTTTCTCTCCACAGCCCCATTA
TGTGG

pTet-Off AIO/uORF nLuc (MluI/XbaI)_stop mutated

ACGCGTTgctcgagcactttggccgcgaatcgatatgtcgagtttactccctatcagtgatagagaacgtatgtcgagtttactccct
atcagtgatagagaacgatgtcgagtttactccctatcagtgatagagaacgtatgtcgagtttactccctatcagtgatagagaacgtat

gtcgagtttactccctatcagtgatagagaacgtatgtcgagtttatccctatcagtgatagagaacgtatgtcgagtttactccctatcagt
gatagagaacgtatgtcgaggtaggcgtgtacggtgggaggcctatataagcaGAGCTCTCTGGCTAACTAGAGAA
CCCACTGCTTACTGGCTTATCGAAATTAATACGACTCACTATAGGcaacaacaacaacaacaacaac
aacaacaacaacaacaacaacaacaacaacaacaacaacaacaacaacaccatggtcaccatggtcaccatggtctacc
aacaacaacaacaaATGGTCTTCACACTCGAAGATTTCGTTGGGGACTGGCGACAGACAGCCG
GCTACAACCTGGACCAAGTCCTTGAACAGGGAGGTGTGTCCAGTTTGTTTCAGAATCTCGG
GGTGTCCGTAACTCCGATCCAAAGGATTGTCCTGAGCGGTGAAAATGGGCTGAAGATCGA
CATCCATGTCATCATCCCGTATGAAGGTCTGAGCGGCGACCAAATGGGCCAGATCGAAAA
AATTTTTAAGGTGGTGTACCCTGTGGATGATCATCACTTTAAGGTGATCCTGCACTATGGCA
CACTGGTAATCGACGGGGTTACGCCGAACATGATCGACTATTTCGGACGGCCGTATGAAG
GCATCGCCGTGTTCGACGGCAAAAAGATCACTGTAACAGGGACCCTGTGGAACGGCAACA
AAATTATCGACGTAAGTATCAAGGTTACAAGACAGGTTTAAGGAGACCAATAGAAACTGGG
CTTGTCGAGACAGAGAAGACTCTTGCGTTTCTGATAGGCACCTATTGGTCTTACTGACATC
CACTTTGCCTTTCTCTCCACAGGAGCGCCTGATCAACCCCGACGGCTCCCTGCTGTTCCG
AGTAACCATCAACGGAGTGACCGGCTGGCGGCTGTGCGAACGCATTCTGGCGGACTACAA
AGACCATGACGGTGATTATAAAGATCATGACATCGATTACAAGGATGACGATGACAAGTAA
GGCCGCGACTCTAGA

pTet-Off AIO/large uORF nLuc (MluI/XbaI)_stop mutated

ACGCGTTgctcgagcactttggccgcgaatcgatatgtcgagtttactccctatcagtgatagagaacgtatgtcgagtttactccct
atcagtgatagagaacgatgtcgagtttactccctatcagtgatagagaacgtatgtcgagtttactccctatcagtgatagagaacgtat
gtcgagtttactccctatcagtgatagagaacgtatgtcgagtttatccctatcagtgatagagaacgtatgtcgagtttactccctatcagt
gatagagaacgtatgtcgaggtaggcgtgtacggtgggaggcctatataagcaGAGCTCTCTGGCTAACTAGAGAA
CCCACTGCTTACTGGCTTATCGAAATTAATACGACTCACTATAGGcaacaacaacaacaacaacaac
aacaacaacaacaacaacaacaacaacaacaacaacaacaacaacaacaccatggtcaccatggtcaccatggtcGCA
GAAATCGGTACTGGCTTTCCATTCGACCCCCATTATGTGGAAGTCCTGGGCGAGCGCATG
CACTACGTCGATGTTGGTCCGCGCGATGGCACCCCTGTGCTGTTCCTGCACGGTAACCCG
ACCTCCTCCTACGTGTGGCGCAACATCATCCCGCATGTTGCACCGACCCATCGCTGCATT
GCTCCAGACCTGATCGGTATGGGCAAATCCGACAAACCAGACCTGGGTTATTTCTTCGACG
ACCACGTCCGCTTCATGGATGCCTTCATCGAAGCCCTGGGTCTGGAAGAGGTCGTCCTGG
TCATTCACGACTGGGGCTCCGCTCTGGGTTTCCACTGGGCCAAGCGCAATCCAGAGCGCG
TCAAAGGTATTGCATTTATGGAGTTCATCCGCCCTATCCCGACCTGGGACGAATGGCCAGA
ATTTGCCCGCGAGACCTTCCAGGCCTTCCGCACCACCGACGTCGGCCGCAAGCTGATCAT
CGATCAGAACGTTTTTATCGAGGGTACGCTGCCGATGGGTGTCGTCCGCCCGCTGACTGA
AGTCGAGATGGACCATTACCGCGAGCCGTTCCTGAATCCTGTTGACCGCGAGCCACTGTG
GCGCTTCCCAAACGAGCTGCCAATCGCCGGTGAGCCAGCGAACATCGTCGCGCTGGTCG
AAGAATACATGGACTGGCTGCACCAGTCCCCTGTCCCGAAGCTGCTGTTCTGGGGCACCC
CAGGCGTTCTGATCCCACCGGCCGAAGCCGCTCGCCTGGCCAAAAGCCTGCCTAACTGCA
AGGCTGTGGACATCGGCCCGGGTCTGAATCTGCTGCAAGAAGACAACCCGGACCTGATCG
GCAGCGAGATCGCGCGCTGGCTGTCGACGCTCGAGATTTCCGGCCACCGGTCGCAACCT
TGGGTACCGCGGGCCCGGGATCCACCGGTCGCAACCTTGGTGAGCAAGGGCGAGGAGCT
GTTCACCGGGGTGGTGCCCATCCTGGTCGAGCTGGACGGCGACGTAAACGGCCACAAGT
TCAGCGTGTCCGGCGAGGGCGAGGGCGATGCCACCTACGGCAAGCTGACCCTGAAGTTC
ATCTGCACCACCGGCAAGCTGCCCGTGCCCTGGCCCACCCTCGTGACCACCCTGACCTAC
GGCGTGCAGTGCTTCAGCCGCTACCCCGACCACATGAAGCAGCACGACTTCTTCAAGTCC
GCCATGCCCGAAGGCTACGTCCAGGAGCGCACCATCTTCTTCAAGGACGACGGCAACTAC
AAGACCCGCGCCGAGGTGAAGTTCGAGGGCGACACCCTGGTGAACCGCATCGAGCTGAA
GGGCATCGACTTCAAGGAGGACGGCAACATCCTGGGGCACAAGCTGGAGTACAACTACAA
CAGCCACAACGTCTATATCATGGCCGACAAGCAGAAGAACGGCATCAAGGTGAACTTCAA
GATCCGCCACAACATCGAGGACGGCAGCGTGCAGCTCGCCGACCACTACCAGCAGAACA
CCCCCATCGGCGACGGCCCCGTGCTGCTGCCCGACAACCACTACCTGAGCACCCAGTCC

GCCCTGAGCAAAGACCCCAACGAGAAGCGCGATCACATGGTCCTGCTGGAGTTCGTGACC
GCCGCCGGGATCACTCTCGGCATGGACGAGCTGTACAAGtaccaacaacaacaacaaATGGTCT
TCACACTCGAAGATTTCGTTGGGGACTGGCGACAGACAGCCGGCTACAACCTGGACCAAG
TCCTTGAACAGGGAGGTGTGTCCAGTTTGTTTCAGAATCTCGGGGTGTCCGTAACTCCGAT
CCAAAGGATTGTCCTGAGCGGTGAAAATGGGCTGAAGATCGACATCCATGTCATCATCCC
GTATGAAGGTCTGAGCGGCGACCAAATGGGCCAGATCGAAAAAATTTTTAAGGTGGTGTAC
CCTGTGGATGATCATCACTTTAAGGTGATCCTGCACTATGGCACACTGGTAATCGACGGGG
TTACGCCGAACATGATCGACTATTTCGGACGGCCGTATGAAGGCATCGCCGTGTTCGACG
GCAAAAAGATCACTGTAACAGGGACCCTGTGGAACGGCAACAAAATTATCGACGTAAGTAT
CAAGGTTACAAGACAGGTTTAAGGAGACCAATAGAAACTGGGCTTGTCGAGACAGAGAAG
ACTCTTGCGTTTCTGATAGGCACCTATTGGTCTTACTGACATCCACTTTGCCTTTCTCTCCA
CAGGAGCGCCTGATCAACCCCGACGGCTCCCTGCTGTTCCGAGTAACCATCAACGGAGTG
ACCGGCTGGCGGCTGTGCGAACGCATTCTGGCGGACTACAAAGACCATGACGGTGATTAT
AAAGATCATGACATCGATTACAAGGATGACGATGACAAGTAAGGCCGCGACTCTAGA
